# Morphology and Phylogeny of *Pestalotiopsis* (*Sporocadaceae*, *Amphisphaeriales*) from *Fagaceae* Leaves in China

**DOI:** 10.1128/spectrum.03272-22

**Published:** 2022-11-10

**Authors:** Ning Jiang, Hermann Voglmayr, Han Xue, Chun-Gen Piao, Yong Li

**Affiliations:** a Key Laboratory of Biodiversity Conservation of National Forestry and Grassland Administration, Ecology and Nature Conservation Institute, Chinese Academy of Forestry, Beijing, China; b Department of Botany and Biodiversity Research, University of Vienna, Vienna, Austria; Agroscope

**Keywords:** new species, phylogeny, plant disease, taxonomy

## Abstract

*Fagaceae* is a family of flowering plants widely distributed in the Northern Hemisphere, including deciduous and evergreen trees and shrubs. Species of *Pestalotiopsis* are well-known agents of leaf spot diseases, but targeted sampling on *Fagaceae* is still missing. To determine the diversity of *Pestalotiopsis* species associated with *Fagaceae* leaf spot in China, investigations were conducted in the main areas of *Fagaceae* distribution from 2016 to 2021. Diseased leaf tissues were collected, and fungal isolates were obtained from leaf spots. In the present study, 43 isolates of *Pestalotiopsis* were studied based on combined morphology and phylogeny. As a result, 10 new species were identified, *viz.*, *Pestalotiopsis anhuiensis*, *P. castanopsidis*, *P. changjiangensis*, *P. cyclobalanopsidis*, *P. foliicola*, *P. guangxiensis*, *P. guizhouensis*, *P. lithocarpi*, *P. shaanxiensis*, and *P. silvicola*, and six new host records were recognized.

**IMPORTANCE** *Pestalotiopsis* is a common fungal genus inhabiting plant tissues as endophytes, pathogens, and saprophytes. *Fagaceae* is a plant family including many important tree species, such as *Castanea mollissima* and *Quercus* spp. In this study, diseased leaves of *Fagaceae* in China were investigated, and 16 *Pestalotiopsis* species were identified based on morphology and phylogeny of combined loci of internal transcribed spacers (ITS), the translation elongation factor 1-α (*tef1*), and the beta-tubulin (*tub2*) genes. Among these, 10 new species were found, and six new host records were revealed. Our study significantly updates the taxonomy of *Pestalotiopsis* and enhances our understanding of leaf diseases of *Fagaceae* hosts.

## INTRODUCTION

*Fagaceae* is an ecologically important family of flowering plants including eight genera and about 927 species worldwide (1). Most species of *Fagaceae* are deciduous trees or shrubs in temperate regions, and some species are evergreens distributed in subtropical to tropical regions. As we know, chestnuts (*Castanea* spp.) provide delicious natural foods, and oaks (*Quercus* spp.) and beeches (*Fagus* spp.) are commonly used as timbers. Many fagaceous species in temperate forests are ecologically highly important as main components of various forest types and provide an important food source for wildlife, and some of them are prominent ornamental trees (2).

More than 320 *Fagaceae* species have been recorded from China, including Chinese chestnut (Castanea mollissima), which provides tree crops, and oriental cork oak (Quercus variabilis), which is widely used as a shade, street, or ornamental tree in China. Leaf spot diseases are common on *Fagaceae* hosts in China, from which several fungal species were revealed based on morphology and phylogeny in recent years (3). For example, Diaporthe eres and Ophiognomonia castaneae cause *Castanea mollissima* brown margin leaf blight in Shandong Province of China (4). Botryosphaeria qinlingensis causes oak frogeye leaf spot disease in China (5). Monochaetia castaneae and 25 other species were reported to be associated with *C. mollissima* leaf diseases in chestnut plantations in China (6).

Pestalotioid fungi are easily characterized by multiseptate and more or less fusiform conidia with appendages at one or both ends, frequently with some melanized cells (7–9). Fungi currently known as pestalotioid fungi are classified in the family *Sporocadaceae*, with 30 accepted genera based on multiple-locus phylogeny and conidial characters (8).

The classification of pestalotioid genera is complicated and has undergone substantial rearrangements in the past decades. The genus *Pestalotia*, originally described by De Notaris (10), contains almost 600 species epithets, the majority of which were over time transferred to separate genera. More than 100 years after its description, in his pioneering revision, Steyaert (11) restricted *Pestalotia* to the generic type species, Pestalotia pezizoides, and separated *Pestalotiopsis* and *Truncatella* based on cell numbers in the conidium: 4 cells in *Truncatella*, 5 cells in *Pestalotiopsis*, and 6 cells in *Pestalotia*. However, Steyaert (11) did not accept *Monochaetia* as a distinct genus but placed its species into *Pestalotiopsis* or *Truncatella* based on cell numbers of the conidium. In contrast, in his revision of *Pestalotia*, Guba (12) did not accept *Pestalotiopsis* and *Truncatella* as distinct genera but re-established *Monochaetia* for species having a single apical and basal appendage, which he placed in three sections based on the number of conidial cells (4, 5, or 6). Subsequently, a hybrid classification scheme was established recognizing all four genera (*Pestalotia*, *Pestalotiopsis*, *Truncatella*, and *Monochaetia*), which was primarily based on the cell numbers per conidium in combination with the number of apical appendages (one versus several). *Monochaetia* was restricted to species with five-celled conidia, while species with four-celled conidia were transferred to *Truncatella* or *Seimatosporium* and species with six-celled conidia to *Seiridium* (13).

Molecular phylogenetic analysis largely confirmed the morphological generic classification of Sutton (13), and it was subsequently further refined. Phylogenetically, *Monochaetia sensu stricto*, *Pestalotiopsis*, *Seiridium*, and *Truncatella* were shown to form distinct clades in *Sporocadaceae* (8, 14), while *Pestalotia* was shown to be synonymous with the older genus *Seiridium* (15). Based on phylogeny of multiple genes and conidial morphology, *Pestalotiopsis sensu lato* was further split into *Pestalotiopsis sensu stricto*, *Neopestalotiopsis*, and *Pseudopestalotiopsis* (16). While *Neopestalotiopsis* can be distinguished from the other genera by a versicolorous medium part of the conidia, *viz.*, a lighter brown second cell and darker brown third and fourth cells, conidia of *Pestalotiopsis* and *Pseudopestalotiopsis* are morphologically indistinguishable but differ molecularly, e.g., by the lengths of their internal transcribed spacer (ITS) sequences (489 to 495 bp in *Pestalotiopsis* versus 536 to 540 bp in *Pseudopestalotiopsis*) (16). Ecologically, species of *Pestalotiopsis sensu lato* are common leaf pathogens infecting various hosts worldwide (17–20).

As *Pestalotiopsis* species are known as leaf spot pathogens on *Fagaceae*, typical spotted leaves were collected to obtain fungal isolates, which were subsequently identified based on both morphological and phylogenetic approaches. The aims of the present study were to reveal hidden species diversity of *Pestalotiopsis* from diseased fagaceous leaves and to evaluate the practicability of host association for species distinction.

## RESULTS

**FIG 1:**
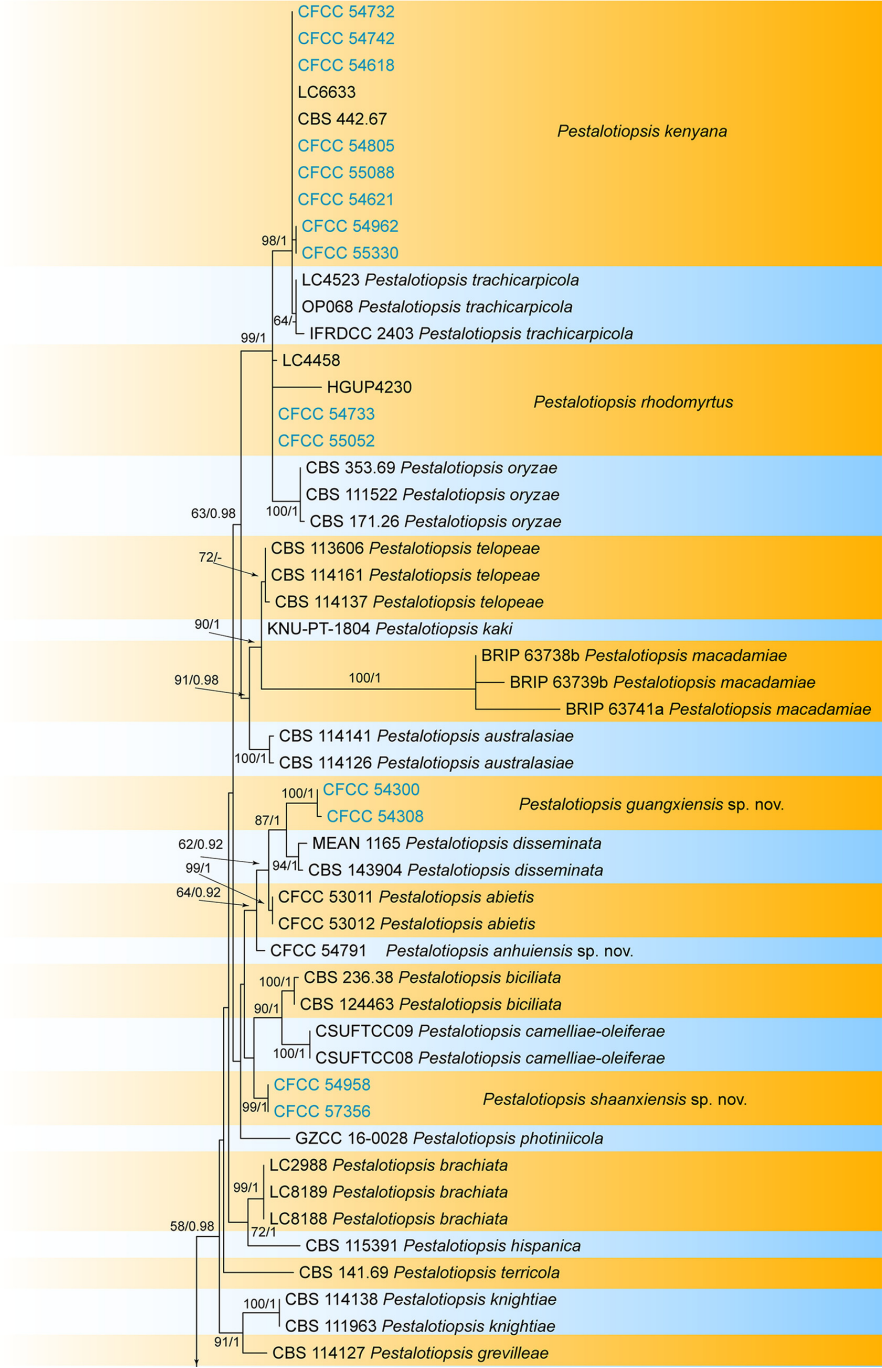

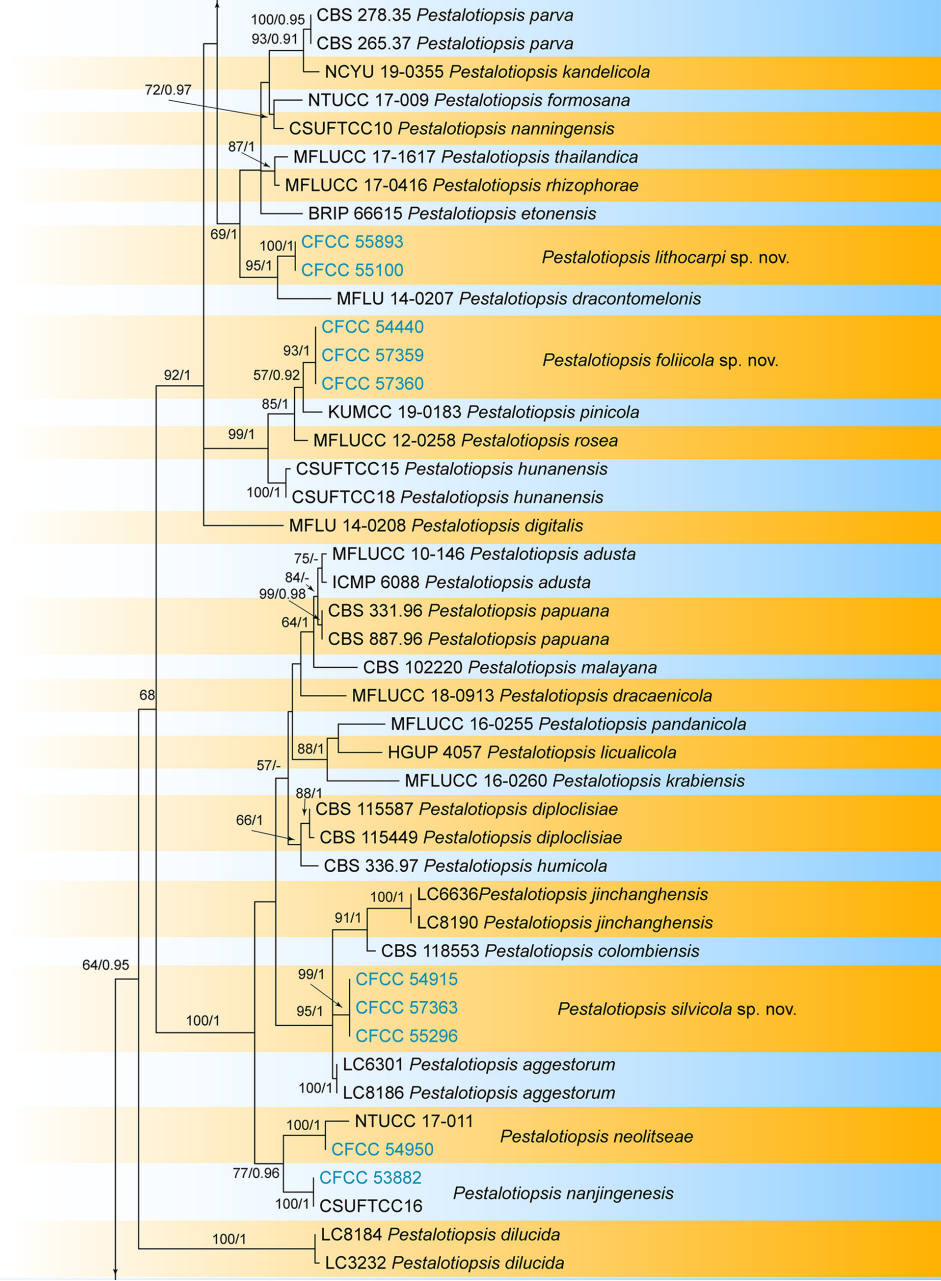

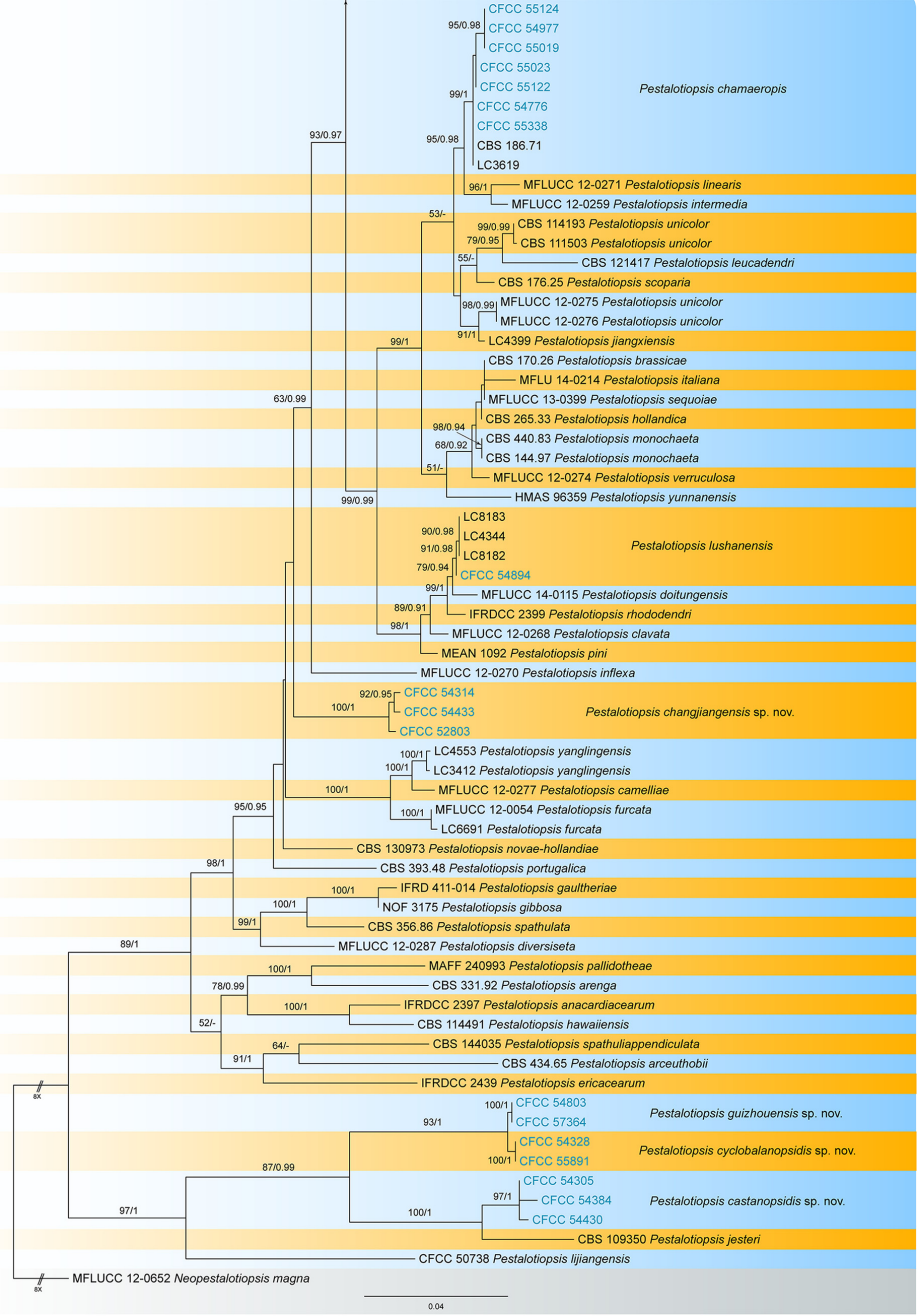
Phylogram of *Pestalotiopsis* resulting from a maximum-likelihood analysis based on a combined matrix of ITS, *tef1*, and *tub2* loci. Numbers above the branches indicate ML bootstrap values (left; values of ≥50% are shown) and Bayesian posterior probabilities (right; values of ≥0.9 are shown). The tree is rooted with Neopestalotiopsis magna (MFLUCC 12–0652). Isolates from the present study are marked in blue.

The combined sequence data set of ITS, *tef1*, and *tub2* comprised 1,552 characters (524 for the ITS, 529 for *tef1*, and 499 for *tub2*) from 162 isolates, including one outgroup taxon, Neopestalotiopsis magna (MFLUCC 12-0652). Of the 1,552 characters included in the phylogenetic analyses, 457 were parsimony informative (84 from the ITS, 200 from *tef1*, and 173 from *tub2*). The best maximum-likelihood (ML) tree (lnL = −12,981.92) revealed by RAxML is shown as a phylogram in Fig. 1. The topologies resulting from ML and Bayesian inference (BI) analyses of the concatenated data set were congruent. Isolates from the present study formed 16 individual clades representing 16 species of *Pestalotiopsis*, including 10 new species (Pestalotiopsis anhuiensis, P. castanopsidis, P. changjiangensis, P. cyclobalanopsidis, P. foliicola, P. guangxiensis, P. guizhouensis, P. lithocarpi, P. shaanxiensis, and P. silvicola) and six known species (P. chamaeropis, P. kenyana, P. lushanensis, P. nanjingensis, P. neolitseae, and P. rhodomyrtus).

## TAXONOMY

**FIG 2:**
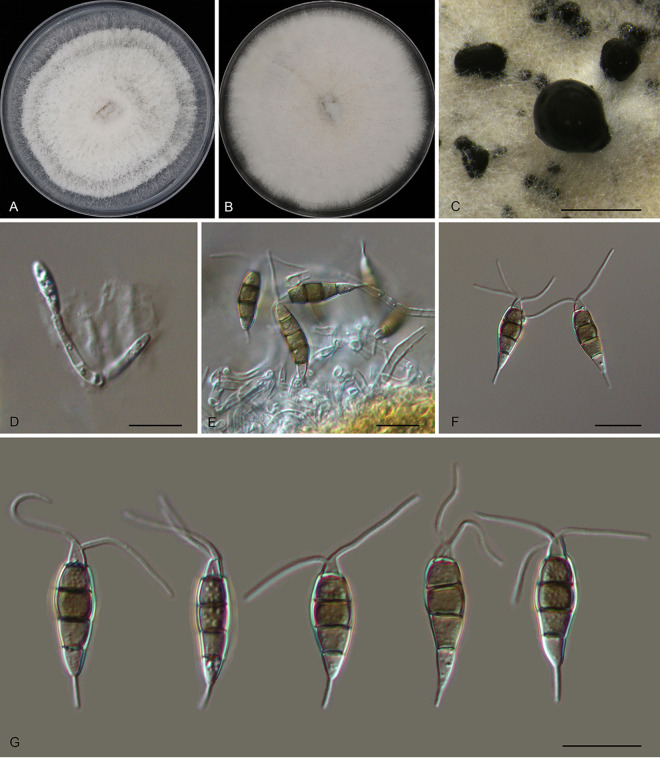
Morphology of *Pestalotiopsis anhuiensis* (CFCC 54791). (A) Colony on PDA after 10 days at 25°C; (B) colony on MEA after 10 days at 25°C; (C) conidiomata formed on PDA; (D and E) conidiogenous cells giving rise to conidia; (F and G) conidia. Bars, 300 μm (C) and 10 μm (D to G).

*Pestalotiopsis anhuiensis* Ning Jiang, sp. nov. (Fig. 2). MycoBank number MB843387. Etymology: named after the collection site of the type specimen, Anhui Province. Typus: China, Anhui Province, Hefei City, Shushan District, Dashushan Forest Park, on diseased leaves of Cyclobalanopsis glauca, 2 November 2019, Dan-ran Bian (holotype CAF 800044; ex-holotype culture CFCC 54791).

Conidiomata in culture sporodochial, aggregated or solitary, erumpent, pulvinate, black, 50 to 300 μm in diameter, exuding black conidial masses. Conidiophores indistinct, usually reduced to conidiogenous cells. Conidiogenous cells hyaline, smooth, cylindrical to spherical, annelidic, 3.5 to 13.5 by 2 to 3.5 μm, mean ± standard deviation (SD) = 8.2 ± 2.6 by 3.1 ± 1.1 μm. Conidia fusoid, straight or slightly curved, 4-septate, smooth, slightly constricted at the septa, (18.5)20.5 to 23.5(25) by (6)6.5 to 7.5(8) μm (measurements of conidia are reported as maximum and minimum in parentheses and the range representing the mean ± standard deviation of the number of measurements given in parentheses), mean ± SD = 21.8 ± 1.4 by 7.1 ± 0.4 μm, length/width ratio (L/W) = 2.7 to 3.8; basal cell obconic with a truncate base, thin-walled, hyaline or pale brown, (3.5)4.5 to 5.5(6) μm; median cells 3, trapezoid or subcylindrical, concolorous, pale brown to brown, thick-walled, the first median cell from base (4)5 to 6 μm long, the second cell 4 to 5 μm long, the third cell (3.5)4 to 5(5.5) μm long, together (12)13 to 15.5(17) μm long; apical cell conic with an acute apex, thin-walled, hyaline, (2.5)3 to 4(4.5) μm long; basal appendage single, unbranched, tubular, centric, straight or slightly bent, (4)4.5 to 6.5(7) μm long, mean ± SD = 5.4 ± 0.9 μm; apical appendages, 2 or 3, unbranched, tubular, centric, straight or bent, (7.5)12.5 to 17.5(20) μm long, mean ± SD = 14.9 ± 2.6 μm. Sexual morph unknown.

Colonies on malt extract agar (MEA) flat, spreading, with flocculent aerial mycelium and entire edge, white, reaching a 70-mm diameter after 10 days at 25°C, forming black conidiomata with black conidial masses; on potato dextrose agar (PDA), flat, spreading, with flocculent aerial mycelium forming concentric rings and entire edge, white, reaching a 70-mm diameter after 10 days at 25°C, forming black conidiomata with black conidial masses.

Notes: *Pestalotiopsis anhuiensis* from *Cyclobalanopsis glauca* is phylogenetically close to Pestalotiopsis abietis, P. disseminata, and *P. guangxiensis* (Fig. 1). Morphologically, *P. anhuiensis* shares similar conidial sizes with *P. abietis* and *P. disseminata* (18.5 to 25 by 6 to 8 μm in *P. anhuiensis* versus 19.9 to 31.2 by 5.8 to 8 μm in *P. abietis* and 18 to 25 by 6.5 to 8 μm in *P. disseminata*) (21–23) and has narrower conidia than *P. guangxiensis* (6 to 8 μm versus 7.5 to 9.5 μm in *P. guangxiensis*) (Table 1). However, *P. anhuiensis* can be distinguished by sequence data (nucleotide differences from *P. abietis*: in the ITS, 1/506 [0.2%]; in *tef1*, 4/470 [0.85%]; in *tub2*, 1/442 (0.23%); nucleotide differences from *P. disseminata*: in the ITS, 3/506 [0.59%], 1 insertion; in *tef1*, 8/470 [1.7%]; in *tub2*, 1 or 2/406 [0.25 to 0.5%]; nucleotide differences from *P. guangxiensis*: in the ITS, 1/506 [0.2%], 1 insertion; in *tef1*, 12/470 [2.55%], 1-bp gap; in *tub2*, 2 or 3/442 [0.45 to 0.68%]).

**TABLE 1:** Synopsis of *Pestalotiopsis* occurring on fagaceous hosts^*a*^

Species	Host(s)	Length of conidia (μm)	Width of conidia (μm)	Length of 3 median cells (μm)	Length of apical appendage (μm)	Length of basal appendage (μm)	Reference
*P. anhuiensis*	*Cyclobalanopsis glauca*	18.5–25	6–8	12–17	7.5–20	4–7	This study
*P. castanopsidis*	*Castanopsis hystrix*, *C. lamontii*	23–29	7–11.5	16–20.5	17–24.5	8.5–15	This study
*P. chamaeropis*	*C. fissa*, *Quercus acutissima*, *Q. aliena*, *Q. variabilis*	20.5–31.5	6.5–9	13–20	8.5–27.5	2.5–12	This study
*P. changjiangensis*	*Castanopsis hainanensis*, *C. tonkinensis*	19–24	7–8.5	13.5–16.5	1.5–7	absent	This study
*P. cyclobalanopsidis*	*Cyclobalanopsis glauca*	18.5–25.5	6–8.5	13–16	5.5–14.5	2–6.5	This study
*P. foliicola*	*Castanopsis faberi*	19.5–24	7–9.5	10.5–16	10.5–37	3–5	This study
*P. guangxiensis*	*Quercus griffithii*	17.5–21	7.5–9.5	12–14	14–19	3–4.5	This study
*P. guizhouensis*	*Cyclobalanopsis glauca*	21–26.5	7–9.5	13–18	7–15	2–8	This study
*P. kenyana*	*Castanea henryi*, *Ca. mollissima*, *Castanopsis fissa*, *C. hystrix*, *Cyclobalanopsis glauca*, *Cy. fleuryi*, *Quercus aliena*, *Q. aliena* var. *acutiserrata*	20.5–28	6–8	NA	3.5–15	1.5–3.5	6; this study
*P. lithocarpi*	*Lithocarpus chiungchungensis*	17–23	5.5–8	12.5–14.5	9–24	2.5–5	This study
*P. lushanensis*	*Quercus serrata*	19.5–26.5	7–9.5	13–17.5	10.5–26.5	2.5–0.5	This study
*P. monochaeta*	Quercus robur	25–42	7–11.5	17–26	40–75	6–14	16
*P. nanjingensis*	*Quercus aliena*	17.5–23.5	6.5–9	13–7.5	8.5–20.5	2–6.5	This study
*P. neolitseae*	*Lithocarpus amygdalifolius*	19–23.5	5.5–7	13–14.5	9.5–14.5	2–3.5	This study
*P. rhodomyrtus*	*Cyclobalanopsis augustinii*, *Quercus aliena*	20–27	6–8	13.5–18	8–17.5	2–7	This study
*P. shaanxiensis*	*Quercus variabilis*	21–25	7–9	13.5–17.5	13–22	1.5–7.5	This study
*P. silvicola*	*Cyclobalanopsis kerrii*	20–26	6–8.5	12–16	12–22.5	3.5–8	This study

a NA, not available.

**FIG 3:**
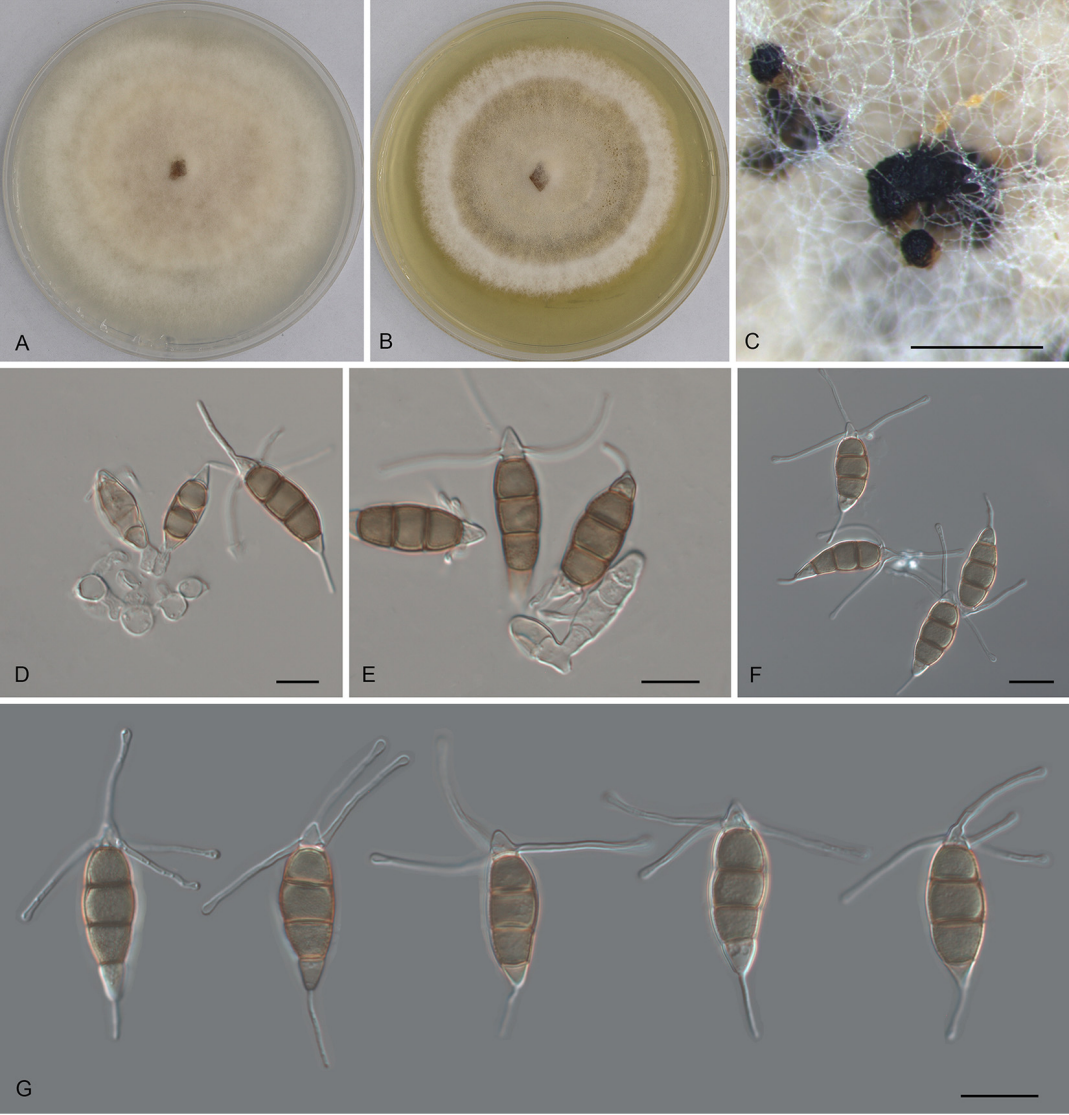
Morphology of *Pestalotiopsis castanopsidis* (CFCC 54430). (A) Colony on PDA after 10 days at 25°C; (B) colony on MEA after 10 days at 25°C; (C) conidiomata formed on PDA; (D and E) conidiogenous cells giving rise to conidia; (F and G) conidia. Bars, 200 μm (C) and 10 μm (D to G).

*Pestalotiopsis castanopsidis* Ning Jiang, sp. nov. (Fig. 3). MycoBank number MB841308. Etymology: named after the host genus, *Castanopsis*. Typus: China, Guangdong Province, Qingyuan City, Yangshan County, Guangdong Nanling Nature Reserve, on diseased leaves of *Castanopsis lamontii*, 4 December 2019, Shang Sun (holotype CAF 800021; ex-holotype culture CFCC 54430).

Conidiomata in culture sporodochial, aggregated or solitary, erumpent, pulvinate, black, 50 to 350 μm in diameter, exuding black conidial masses. Conidiophores indistinct, usually reduced to conidiogenous cells. Conidiogenous cells hyaline, smooth, cylindrical to spherical, annelidic, 5 to 11.5 by 2.5 to 7 μm, mean ± SD = 7.2 ± 2.5 by 4.4 ± 1.4 μm. Conidia fusoid, straight or slightly curved, 4-septate, smooth, slightly constricted at the septa, (23)24 to 27.5(29) by (7)8 to 11(11.5) μm, mean ± SD = 25.7 ± 1.8 by 9.3 ± 1.4 μm, L/W = 2.3 to 4; basal cell obconic with a truncate base, thin-walled, hyaline or pale brown, 4 to 5 μm; median cells, 3, trapezoid or subcylindrical, concolorous, brown, thick-walled, the first median cell from base (5.5)6 to 7 μm long, the second cell (5.5)6 to 7.5(8) μm long, the third cell 5.5 to 6.5(7) μm long, together (16)17 to 20(20.5) μm long; apical cell conic with an acute apex, thin-walled, hyaline, (2.5)3 to 4.5(5) μm long; basal appendage single, unbranched, tubular, centric, straight or slightly bent, (8.5)9.5 to 14(15) μm long, mean ± SD = 11.7 ± 2.4 μm; apical appendages, 3 or 4, unbranched, tubular, knobbed, centric, straight or slightly bent, (17)17.5 to 23(24.5) μm long, mean ± SD = 20.3 ± 2.7 μm. Sexual morph unknown.

Colonies on MEA flat, spreading, with flocculent aerial mycelium forming concentric rings and entire edge, off-white to sienna, reaching 60 mm diameter after 10 days at 25°C, forming black conidiomata with black conidial masses; on PDA, flat, spreading, with flocculent aerial mycelium forming concentric rings and undulate edge, pale luteous to fawn, reaching a 70-mm diameter after 10 days at 25°C, forming black conidiomata with black conidial masses.

Additional materials, China, Guangdong Province, Shaoguan City, Lechang City, Dayaoshan Forest Farm, on diseased leaves of Castanopsis hystrix, 4 December 2019, Dan-ran Bian (CFCC 54305 and CFCC 54384).

Notes: Three isolates of *Pestalotiopsis castanopsidis* from *Castanopsis hystrix* and C. lamontii clustered into a distinct clade phylogenetically close to P. jesteri (Fig. 1). However, *P. castanopsidis* differs from *P. jesteri* by obviously larger conidia (23 to 29 by 7 to 11.5 μm in *P. castanopsidis* versus 19 to 23 by 5 to 7 μm in *P. jesteri*) (24). In addition, *P. castanopsidis* can be distinguished from *P. jesteri* by sequence data (nucleotide differences: in the ITS, 4/364 [1.1%]; in *tub2*, 14 or 15/438 [3.2 to 3.42%]).

*Pestalotiopsis chamaeropis* S. S. Maharachch, K. D. Hyde & P. W. Crous, Studies in Mycology 79:158 (2014).

Conidiomata in culture sporodochial, solitary, erumpent, pulvinate, black, 150 to 450 μm in diameter, exuding black conidial masses. Conidiophores indistinct, usually reduced to conidiogenous cells. Conidiogenous cells hyaline, smooth, cylindrical to subcylindrical, annelidic, 4.5 to 12 by 2.5 to 4.5 μm, mean ± SD = 6.9 ± 1.5 by 3.6 ± 0.7 μm. Conidia fusoid, straight or slightly curved, 4-septate, smooth, slightly constricted at the septa, (20.5)23 to 28.5(31.5) by (6.5)7.5 to 8.5(9) μm, mean ± SD = 25.8 ± 2.6 by 8 ± 0.7 μm, L/W = 2.5 to 4.4; basal cell obconic with a truncate base, thin-walled, hyaline or pale brown, (3.5)4.5 to 7(8) μm; median cells 3, trapezoid or subcylindrical, concolorous, brown, thick-walled, the first median cell from base (4)4.5 to 6(7) μm long, the second cell (4.5)5 to 6.5(7) μm long, the third cell (4)5 to 6(6.5) μm long, together (13)15 to 18(20) μm long; apical cell conic with an acute apex, thin-walled, hyaline, (3)3.5 to 5(5.5) μm long; basal appendage unbranched, tubular, centric, straight or slightly bent, (2.5)4 to 8.5(12) μm long, mean ± SD = 6.3 ± 2.2 μm; apical appendages, 2 or 3, unbranched, tubular, centric, straight or slightly bent, (8.5)12 to 21.5(27.5) μm long, mean ± SD = 16.8 ± 4.8 μm. Sexual morph unknown.

Colonies on MEA flat, spreading, with flocculent aerial mycelium forming concentric rings and entire edge, white, reaching a 70-mm diameter after 10 days at 25°C, forming black conidiomata with black conidial masses; on PDA, flat, spreading, with flocculent aerial mycelium forming concentric rings and entire edge, white to buff, reaching a 70-mm diameter after 10 days at 25°C, forming black conidiomata with black conidial masses.

Materials examined, China, Anhui Province, Hefei City, Shushan District, Dashushan Forest Park, on diseased leaves of Quercus aliena, 2 November 2019, Dan-ran Bian (CFCC 55019 and CFCC 55122); Anhui Province, Hefei City, Shushan District, Dashushan Forest Park, on diseased leaves of Quercus acutissima, 2 November 2019, Dan-ran Bian (CFCC 54977); Anhui Province, Hefei City, Shushan District, Dashushan Forest Park, on diseased leaves of *Quercus variabilis*, 2 November 2019, Dan-ran Bian (CFCC 54776); Guangdong Province, Shaoguan City, Lechang City, Dayaoshan Forest Farm, on diseased leaves of Castanopsis fissa, 4 December 2019, Shang Sun (CFCC 55023); Shaanxi Province, Xian City, Zhouzhi County, Heihe Forest Park, on diseased leaves of *Quercus variabilis*, 6 September 2019, Yong Li (CFCC 55338); Sichuan Province, Yaan City, Shimian County, on diseased leaves of Quercus acutissima, 10 September 2020, Ning Jiang (CFCC 55124).

Notes: Seven new isolates of *Pestalotiopsis chamaeropis* were collected from four fagaceous hosts, forming a well-supported clade with the ex-type strain CBS 186.71 (Fig. 1). In addition, samples from the present study agree well with the ex-type strain in conidial dimension and morphological characters (21 to 28 by 6 to 9.5 μm in CBS 186.71) (16). Hence, Castanopsis fissa, *Quercus acutissima*, *Q. aliena*, and *Q. variabilis* become new hosts for *Pestalotiopsis chamaeropis*, which was originally described from Chamaerops humilis (16).

**FIG 4:**
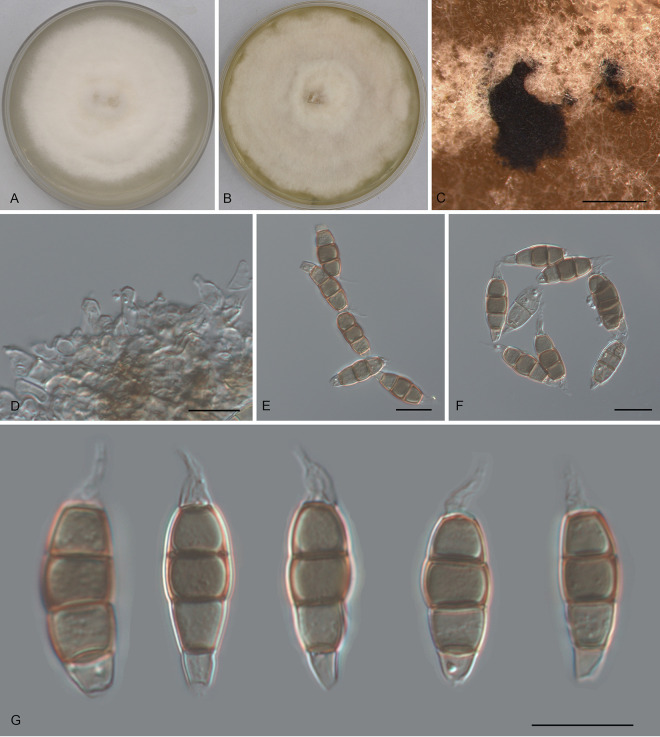
Morphology of *Pestalotiopsis changjiangensis* (CFCC 54314). (A) Colony on PDA after 10 days at 25°C; (B) colony on MEA after 10 days at 25°C; (C) conidioma formed on PDA; (D) conidiogenous cells; (E to G) conidia. Bars, 300 μm (C) and 10 μm (D to G).

*Pestalotiopsis changjiangensis* Ning Jiang, sp. nov. (Fig. 4). MycoBank number MB841309. Etymology: named after the collection site of the type specimen, Changjiang Li Autonomous County. Typus: China, Hainan Province, Changjiang Li Autonomous County, Bawangling National Forest Park, on diseased leaves of Castanopsis tonkinensis, 16 November 2018, Yong Li (holotype CAF 800024; ex-holotype culture CFCC 54314).

Conidiomata in culture sporodochial, aggregated or solitary, erumpent, pulvinate, dark brown, 300 to 850 μm in diameter, exuding black conidial masses. Conidiophores indistinct, usually reduced to conidiogenous cells. Conidiogenous cells hyaline, smooth, cylindrical to spherical, annelidic, 3.5 to 6 by 2 to 5.5 μm, mean ± SD = 4.7 ± 1.1 by 3.4 ± 1 μm. Conidia fusoid, straight, 4-septate, smooth, not constricted or slightly constricted at the septa, (19)20 to 22.5(24) by 7 to 8(8.5) μm, mean ± SD = 21.2 ± 1.4 by 7.7 ± 0.4 μm, L/W = 2.4 to 3.3; basal cell obconic with a truncate base, thin-walled, hyaline or pale brown, (2)3 to 4 μm; median cells 3, trapezoid or subcylindrical, pale brown to brown, thick-walled, the first median cell from base 4.5 to 5.5 μm long, the second cell (4.5)5 to 5.5 μm long, the third cell (4)4.5 to 6 μm long, together (13.5)14.5 to 16(16.5) μm long; apical cell conic with an acute apex, thin-walled, hyaline or pale brown, (2)2.5 to 4(5) μm long; basal appendage indistinct or absent; apical appendage indistinct, tubular, bent, 1.5 to 4.5(7) μm long, mean ± SD = 3 ± 1.1 μm. Sexual morph unknown.

Colonies on MEA flat, spreading, with flocculent aerial mycelium and undulate edge, pale luteous, reaching a 70-mm diameter after 10 days at 25°C, sterile; on PDA, flat, spreading, with flocculent aerial mycelium forming concentric rings and undulate edge, white, reaching a 65-mm diameter after 10 days at 25°C, forming black conidiomata with black conidial masses.

Additional materials examined, China, Hainan Province, Changjiang Li Autonomous County, Bawangling National Forest Park, on diseased leaves of Castanopsis hainanensis, 14 November 2018, Yong Li (CFCC 54433); Hainan Province, Changjiang Li Autonomous County, Bawangling National Forest Park, on diseased leaves of Cyclobalanopsis austrocochinchinensis, 16 November 2018, Yong Li (CFCC 52803).

Notes: Three isolates of *Pestalotiopsis* collected from *Castanopsis hainanensis*, *C. tonkinensis*, and *Cyclobalanopsis austrocochinchinensis* clustered into a distinct and well-supported clade, which is newly described as *P. changjiangensis* here (Fig. 1). Its conidia are characterized by the lack of a basal appendage and an indistinct apical appendage, which is unique within the genus *Pestalotiopsis*.

**FIG 5:**
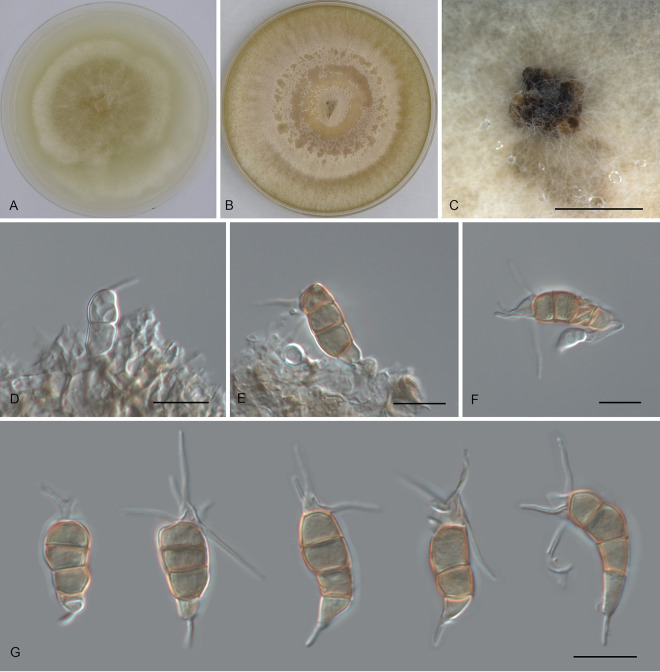
Morphology of *Pestalotiopsis cyclobalanopsidis* (CFCC 54328). (A) Colony on PDA after 10 days at 25°C; (B) colony on MEA after 10 days at 25°C; (C) conidioma formed on PDA; (D to F) conidiogenous cells giving rise to conidia; (G) conidia. Bars, 500 μm (C) and 10 μm (D to G).

*Pestalotiopsis cyclobalanopsidis* Ning Jiang, sp. nov. (Fig. 5). MycoBank number MB841310. Etymology: named after the host genus, *Cyclobalanopsis*. Typus: China, Guangdong Province, Shaoguan City, Lechang City, Dayaoshan Forest Farm, on diseased leaves of *Cyclobalanopsis glauca*, 4 December 2019, Shang Sun (holotype CAF 800022; ex-holotype culture CFCC 54328).

Conidiomata in culture sporodochial, solitary, erumpent, pulvinate, dark brown, 250 to 700 μm in diameter, exuding black conidial masses. Conidiophores indistinct, usually reduced to conidiogenous cells. Conidiogenous cells hyaline, smooth, cylindrical to spherical, annelidic, 6 to 8 by 2 to 4.5 μm, mean ± SD = 7.2 ± 0.9 by 3.6 ± 0.8 μm. Conidia fusoid, curved, 4-septate, smooth, slightly constricted at the septa, (18.5)20 to 24.5(25.5) by (6)6.5 to 8(8.5) μm, mean ± SD = 22.3 ± 1.3 by 7.3 ± 0.5 μm, L/W = 2.6 to 3.6; basal cell obconic with a truncate base, thin-walled, hyaline to pale brown, 4 to 6(6.5) μm; median cells 3, trapezoid or subcylindrical, concolorous, brown, thick-walled, the first median cell from base 4.5 to 6(6.5) μm long, the second cell (3.5)4 to 5 μm long, the third cell (4)4.5 to 5.5 μm long, together 13 to 15.5(16) μm long; apical cell conic with an acute apex, thin-walled, hyaline to pale brown, 3 to 4 μm long; basal appendage unbranched, tubular, centric, straight, (2)2.5 to 5.5(6.5) μm long, mean ± SD = 3.8 ± 1.5 μm; apical appendages, 2 to 5, unbranched, tubular, bent, (5.5)7 to 13(14.5) μm long, mean ± SD = 10.1 ± 3 μm. Sexual morph unknown.

Colonies on MEA flat, spreading, with flocculent aerial mycelium forming concentric rings and entire, hazel to off-white, reaching a 70-mm diameter after 10 days at 25°C, sterile; on PDA, flat, spreading, with flocculent aerial mycelium forming concentric rings and undulate edge, white to luteous, reaching a 50-mm diameter after 10 days at 25°C, forming dark brown conidiomata with black conidial masses.

Additional material examined, China, Guangdong Province, Shaoguan City, Lechang City, Dayaoshan Forest Farm, on diseased leaves of *Cyclobalanopsis glauca*, 4 December 2019, Shang Sun (CFCC 55891).

Notes: *Pestalotiopsis cyclobalanopsidis* is closely related to *P. castanopsidis*, *P. guizhouensis*, and *P. jesteri* (Fig. 1). However, *P. cyclobalanopsidis* differs from them by distinctly curved conidia (24).

**FIG 6:**
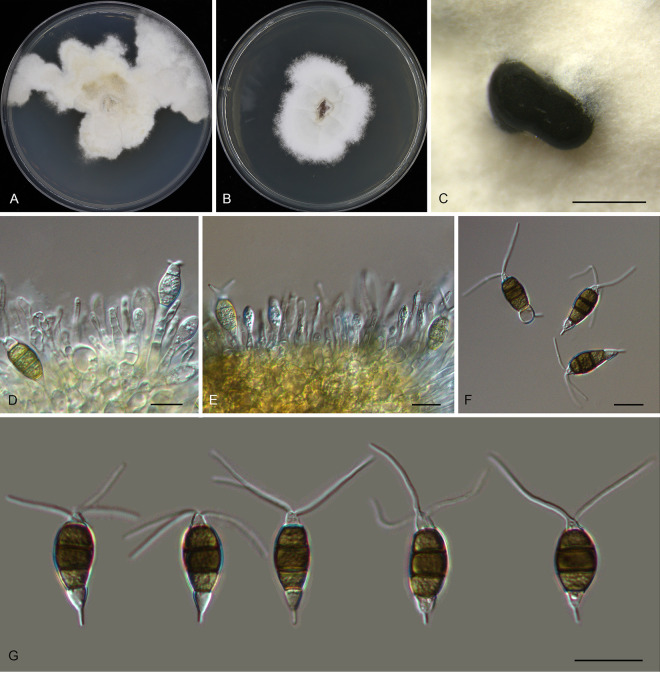
Morphology of *Pestalotiopsis foliicola* (CFCC 54440). (A) Colony on PDA after 10 days at 25°C; (B) colony on MEA after 10 days at 25°C; (C) conidioma formed on PDA; (D and E) conidiogenous cells giving rise to conidia; (F and G) conidia. Bars, 500 μm (C) and 10 μm (D to G).

*Pestalotiopsis foliicola* Ning Jiang, sp. nov. (Fig. 6). MycoBank number MB843388. Etymology: *folium* = “leaf” and *-cola* = “inhabiting”; in reference to substrate origin of the type strain, leaves. Typus: China, Jiangxi Province, Xinyu City, Fenyi County, Dagangshan Nature Reserve, on diseased leaves of Castanopsis faberi, 13 November 2019, Shang Sun (holotype CAF 800045; ex-holotype culture CFCC 54440).

Conidiomata in culture sporodochial, aggregated or solitary, erumpent, pulvinate, black, 100 to 450 μm in diameter, exuding black conidial masses. Conidiophores indistinct, usually reduced to conidiogenous cells. Conidiogenous cells hyaline, smooth, cylindrical to ampulliform, annelidic, 6.5 to 20 by 2.5 to 6.5 μm, mean ± SD = 12.7 ± 4.2 by 3.1 ± 1.2 μm. Conidia fusoid, straight or slightly curved, 4-septate, smooth, slightly constricted at the septa, (19.5)20 to 23(24) by (7)7.5 to 9(9.5) μm, mean ± SD = 21.5 ± 1.5 by 8.5 ± 0.5 μm, L/W = 2.1 to 2.9; basal cell obconic with a truncate base, thin-walled, hyaline or pale brown, (3)3.5 to 5(6) μm; median cells 3, trapezoid or subcylindrical, concolorous, pale brown to brown, thick-walled, the first median cell from base (4)4.5 to 5.5(6) μm long, the second cell (4)4.5 to 5.5(6) μm long, the third cell (3)4.5 to 5.5(6) μm long, together (10.5)13.5 to 15.5(16) μm long; apical cell conic with an acute apex, thin-walled, hyaline or pale brown, (2.5)3 to 4(4.5) μm long; basal appendage single, unbranched, tubular, centric, straight or slightly bent, (3)3.5 to 4.5(5) μm long, mean ± SD = 3.7 ± 0.7 μm; apical appendages, 2 or 3, unbranched, tubular, centric, straight or bent, (10.5)14 to 26(37) μm long, mean ± SD = 20 ± 5.8 μm. Sexual morph unknown.

Colonies on MEA flat, with flocculent aerial mycelium and crenate edge, white, reaching a 35-mm diameter after 10 days at 25°C, sterile; on PDA, flat, spreading, with flocculent aerial mycelium and irregular edge, white to isabelline, reaching a 40-mm diameter after 10 days at 25°C, forming black conidiomata with black conidial masses.

Additional materials examined, China, Jiangxi Province, Xinyu City, Fenyi County, Dagangshan Nature Reserve, on diseased leaves of *Castanopsis faberi*, 13 November 2019, Shang Sun (CFCC 57359 and CFCC 57360).

Notes: Three isolates of *Pestalotiopsis foliicola* from *Castanopsis faberi* clustered into a distinct clade phylogenetically close to P. pinicola and P. rosea (Fig. 1). However, *P. foliicola* differs from *P. pinicola* and *P. rosea* by wider conidia (7 to 9.5 μm in *P. foliicola* versus 5 to 7 μm in *P. pinicola* and 5.7 to 7 μm in *P. rosea*) (25, 26).

**FIG 7:**
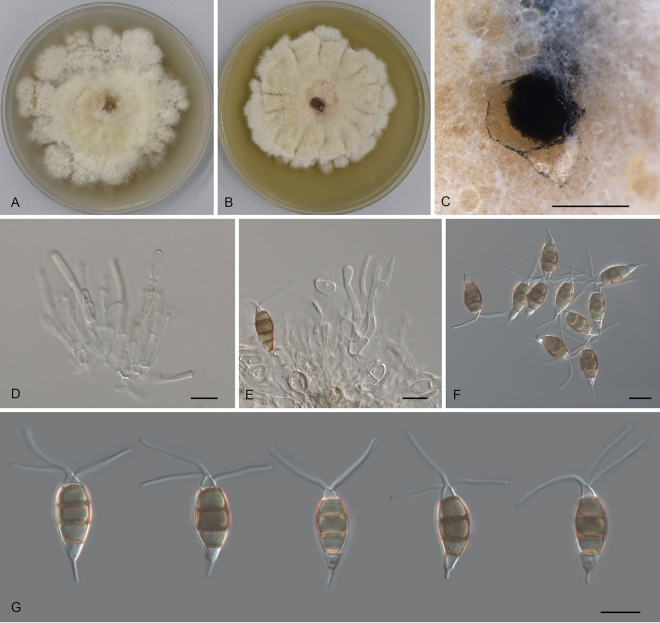
Morphology of *Pestalotiopsis guangxiensis* (CFCC 54308). (A) Colony on PDA after 10 days at 25°C; (B) colony on MEA after 10 days at 25°C; (C) conidioma formed on PDA; (D and E) conidiogenous cells giving rise to conidia; (F and G) conidia. Bars, 500 μm (C) and 10 μm (D to G).

*Pestalotiopsis guangxiensis* Ning Jiang, sp. nov. (Fig. 7). MycoBank number MB841311. Etymology: named after the collection site of the type specimen, Guangxi Zhuang Autonomous Region. Typus: China, Guangxi Zhuang Autonomous Region, Nanning City, Qingxiushan District, Qingxiushan Park, on diseased leaves of Quercus griffithii, 4 December 2019, Dan-ran Bian (holotype CAF 800023; ex-holotype culture CFCC 54308).

Conidiomata in culture sporodochial, aggregated or solitary, erumpent, pulvinate, black, 350 to 800 μm in diameter, exuding black conidial masses. Conidiophores indistinct, usually reduced to conidiogenous cells. Conidiogenous cells hyaline, smooth, cylindrical to subcylindrical, annelidic, 8.5 to 17.5 by 2 to 5 μm, mean ± SD = 11.4 ± 2.7 by 3.1 ± 1.3 μm. Conidia fusoid, straight or slightly curved, 4-septate, smooth, slightly constricted at the septa, (17.5)18 to 20.5(21) by (7.5)8 to 9(9.5) μm, mean ± SD = 19.2 ± 1.2 by 8.3 ± 0.5 μm, L/W = 1.9 to 2.6; basal cell obconic with a truncate base, thin-walled, hyaline, 2.5 to 4.5 μm; median cells 3, trapezoid or subcylindrical, concolorous, brown, thick-walled, the first median cell from base 4 to 4.5 μm long, the second cell 4 to 5(5.5) μm long, the third cell 4 to 5 μm long, together (12)12.5 to 13.5(14) μm long; apical cell conic with an acute apex, thin-walled, hyaline, (2)2.5 to 3(3.5) μm long; basal appendage unbranched, tubular, centric, straight, (3)3.5 to 4(4.5) μm long, mean ± SD = 4 ± 0.5 μm; apical appendages, 2 or 3, unbranched, tubular, centric, straight or slightly bent, (14)15 to 18.5(19) μm long, mean ± SD = 16.8 ± 1.9 μm. Sexual morph unknown.

Colonies on MEA flat, spreading, with flocculent aerial mycelium forming radially folded surface and undulate edge, isabelline, reaching a 55-mm diameter after 10 days at 25°C, producing yellow droplet, sterile; on PDA, flat, spreading, with flocculent aerial mycelium and crenate edge, white to fawn, reaching a 65-mm diameter after 10 days at 25°C, forming black conidiomata with black conidial masses.

Additional material examined, China, Guangxi Zhuang Autonomous Region, Nanning City, Qingxiushan District, Qingxiushan Park, on diseased leaves of *Quercus griffithii*, 4 December 2019, Dan-ran Bian (CFCC 54300).

Notes: Two isolates of *P. guangxiensis* from *Quercus griffithii* formed a well-supported clade phylogenetically close to *P. disseminata* (Fig. 1). *P. guangxiensis* can be distinguished from *P. disseminata* by wider conidia (7.5 to 9.5 μm in *P. guangxiensis* versus 6.5 to 8 μm in *P. disseminata*) (21, 23). Additionally, *P. guangxiensis* differs from *P. disseminata* by sequence data (nucleotide differences: in the ITS, 4/506 [0.8%], 1 insertion; in *tef1*, 7/471 [1.49%], 6 insertions; in *tub2*, 1 to 3/406 [0.25 to 0.74%]).

**FIG 8:**
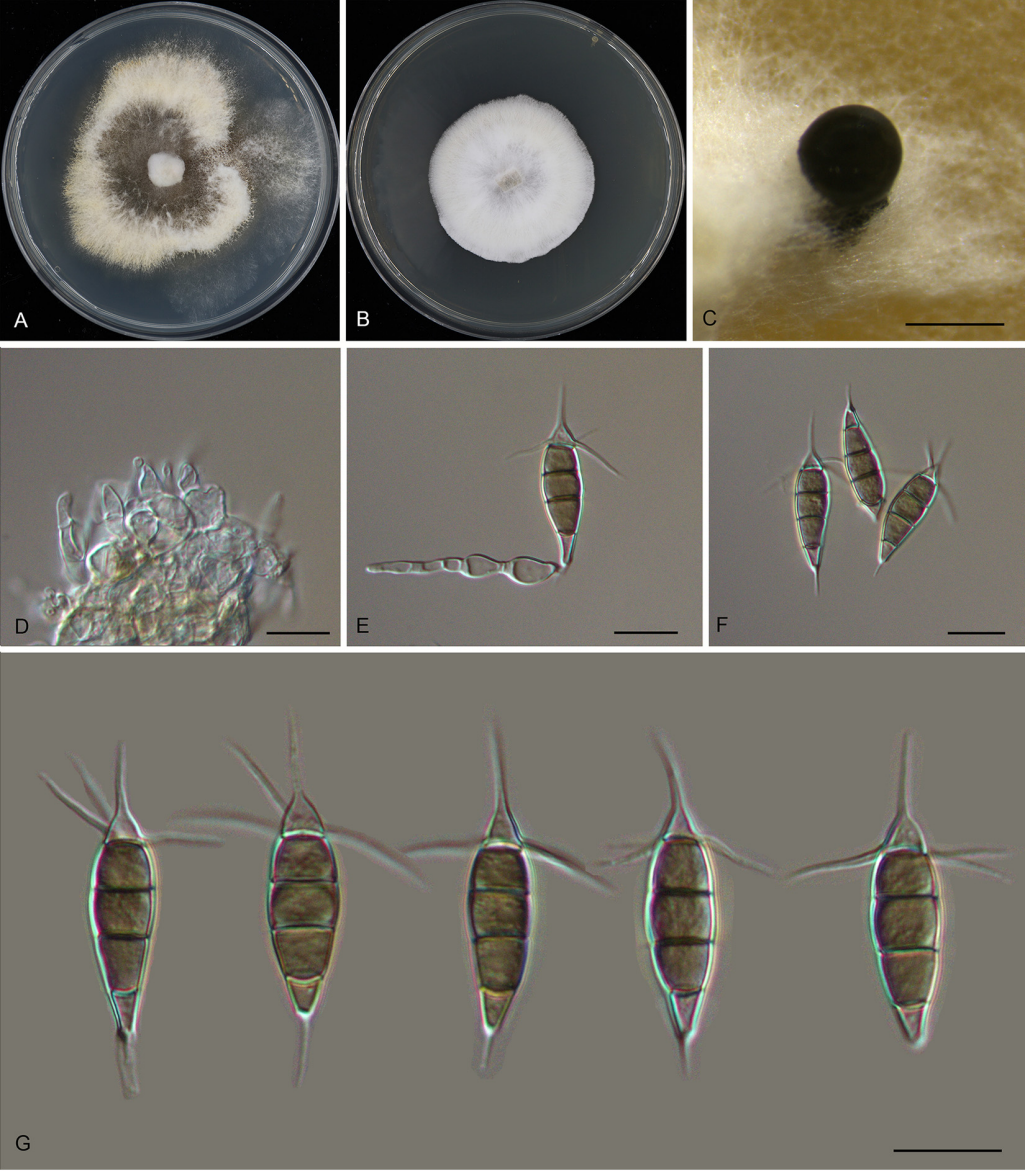
Morphology of *Pestalotiopsis guizhouensis* (CFCC 54803). (A) Colony on PDA after 10 days at 25°C; (B) colony on MEA after 10 days at 25°C; (C) conidioma formed on PDA; (D and E) conidiogenous cells giving rise to conidia; (F and G) conidia. Bars, 300 μm (C) and 10 μm (D to G).

*Pestalotiopsis guizhouensis* Ning Jiang, sp. nov. (Fig. 8). MycoBank number MB843389. Etymology: named after the collection site of the type specimen, Guizhou Province. Typus: China, Guizhou Province, Zunyi City, Suiyang County, Kuankuoshui Natural Reserve, on diseased leaves of *Cyclobalanopsis glauca*, 23 November 2019, Dan-ran Bian (holotype CAF 800046; ex-holotype culture CFCC 54803).

Conidiomata in culture sporodochial, aggregated or solitary, erumpent, pulvinate, black, 100 to 400 μm in diameter, exuding black conidial masses. Conidiophores indistinct, usually reduced to conidiogenous cells. Conidiogenous cells hyaline, smooth, ampulliform to spherical, annelidic, 6 to 17.5 by 3 to 6 μm, mean ± SD = 9.6 ± 4.3 by 4.5 ± 0.9 μm. Conidia fusoid, straight or slightly curved, 4-septate, smooth, slightly constricted at the septa, (21)22.5 to 25.5(26.5) by (7)7.5 to 8.5(9.5) μm, mean ± SD = 23.8 ± 1.6 by 8.1 ± 0.6 μm, L/W = 2.4 to 3.5; basal cell obconic with a truncate base, thin-walled, hyaline or pale brown, (2.5)3.5 to 5.5(6.5) μm; median cells 3, trapezoid or subcylindrical, concolorous, pale brown to brown, thick-walled, the first median cell from base (4.5)5 to 7(7.5) μm long, the second cell (3.5)4.5 to 5.5(6) μm long, the third cell (4)4.5 to 5.5(6) μm long, together (13)14.5 to 17.5(18) μm long; apical cell conic with an acute apex, thin-walled, hyaline, (2.5)3 to 4.5(5) μm long; basal appendage single, unbranched, tubular, centric, straight or slightly bent, (2)3.5 to 6.5(8) μm long, mean ± SD = 4.8 ± 1.5 μm; apical appendages, 3 or 4, unbranched, tubular, attenuate toward the apex, centric, straight or slightly bent, (7)8 to 12(15) μm long, mean ± SD = 10.2 ± 2 μm. Sexual morph unknown.

Colonies on MEA flat, spreading, with flocculent aerial mycelium and entire edge, white, reaching a 35-mm diameter after 10 days at 25°C, sterile; on PDA, flat, spreading, with flocculent aerial mycelium and irregular edge, sienna, reaching a 55-mm diameter after 10 days at 25°C, forming black conidiomata with black conidial masses.

Additional material examined, China, Guizhou Province, Zunyi City, Suiyang County, Kuankuoshui Natural Reserve, on diseased leaves of *Cyclobalanopsis glauca*, 23 November 2019, Dan-ran Bian (CFCC 57364).

Notes: Two isolates of *Pestalotiopsis guizhouensis* from *Cyclobalanopsis glauca* clustered into a distinct clade phylogenetically close to *P. cyclobalanopsidis* (Fig. 1). However, *P. guizhouensis* differs from *P. cyclobalanopsidis* by conidial shape (straight or slightly curved conidia in *P. guizhouensis* versus distinctly curved conidia in *P. cyclobalanopsidis*).

*Pestalotiopsis kenyana* S. S. Maharachch, K. D. Hyde & P. W. Crous, Studies in Mycology 79:158 (2014).

Conidiomata in culture sporodochial, solitary, erumpent, pulvinate, black, 150 to 650 μm in diameter, exuding black conidial masses. Conidiophores indistinct, usually reduced to conidiogenous cells. Conidiogenous cells hyaline, smooth, cylindrical to subcylindrical, annelidic, 5.5 to 15.5 by 2 to 4.5 μm, mean ± SD = 8.9 ± 1.4 by 3.3 ± 0.9 μm. Conidia fusoid, straight or slightly curved, 4-septate, smooth, slightly constricted at the septa, (21)24 to 28.5(30) by (6)6.5 to 8(8.5) μm, mean ± SD = 26.1 ± 2.3 by 7.3 ± 0.5 μm, L/W = 2.6 to 4.9; basal cell obconic with a truncate base, thin-walled, hyaline or pale brown, (4)4.5 to 6.5(8.5) μm; median cells 3, trapezoid or subcylindrical, concolorous, brown, thick-walled, the first median cell from base (4.5)5 to 6(6.5) μm long, the second cell (4.5)5 to 6(8) μm long, the third cell (4.5)5 to 6(6.5) μm long, together (13.5)15 to 17.5(19) μm long; apical cell conic with an acute apex, thin-walled, hyaline, (3)4 to 5.5(6.5) μm long; basal appendage unbranched, tubular, centric, straight or slightly bent, (2.5)4 to 6.5(8.5) μm long, mean ± SD = 5.2 ± 1.5 μm; apical appendages, 2 or 3, unbranched, tubular, centric, straight or slightly bent, (4)9 to 15(20) μm long, mean ± SD = 12.2 ± 3.1 μm. Sexual morph unknown.

Colonies on MEA flat, spreading, with flocculent aerial mycelium forming concentric rings and entire edge, white to pale luteous, reaching a 70-mm diameter after 10 days at 25°C, forming black conidiomata with black conidial masses; on PDA, flat, spreading, with flocculent aerial mycelium and entire edge, white to luteous, reaching a 70-mm diameter after 10 days at 25°C, forming black conidiomata with black conidial masses.

Materials examined: China, Henan Province, Xinyang City, Shihe District, Jigong Mountain, on diseased leaves of Cyclobalanopsis fleuryi, 7 August 2019, Yong Li (CFCC 55330); Henan Province, Xinyang City, Shihe District, Jigong Mountain, on diseased leaves of Cyclobalanopsis neglecta, 7 August 2019, Yong Li (CFCC 54732); Henan Province, Xinyang City, Shihe District, Jigong Mountain, on diseased leaves of Castanopsis fissa, 7 August 2019, Yong Li (CFCC 55088); Guangdong Province, Qingyuan City, Yangshan County, Guangdong Nanling Nature Reserve, on diseased leaves of *Castanopsis hystrix*, 4 December 2019, Shang Sun (CFCC 54742); Guangdong Province, Qingyuan City, Yangshan County, Guangdong Nanling Nature Reserve, on diseased leaves of *Cyclobalanopsis glauca*, 4 December 2019, Shang Sun (CFCC 54805); Guizhou Province, Zunyi City, Suiyang County, Kuankuoshui Natural Reserve, on diseased leaves of *Quercus aliena*, 23 November 2019, Dan-ran Bian (CFCC 54962); Guizhou Province, Zunyi City, Suiyang County, Kuankuoshui Natural Reserve, on diseased leaves of *Quercus aliena* var. *acutiserrata*, 23 November 2019, Dan-ran Bian (CFCC 54621 and CFCC 54618).

Notes: Eight new isolates of *Pestalotiopsis kenyana* were collected from six species and a variety of *Fagaceae*, which agree well with the ex-type strain CBS 442.67 in conidial dimension and characters (22 to 29 by 7 to 9 μm) (16). Hence, *Castanopsis fissa*, *C. hystrix*, *Cyclobalanopsis fleuryi*, *Cy. glauca*, *Cy. neglecta*, *Quercus aliena*, and *Q. aliena* var. *acutiserrata* become new hosts for *Pestalotiopsis kenyana*, which was originally described from *Coffea* species (16).

**FIG 9:**
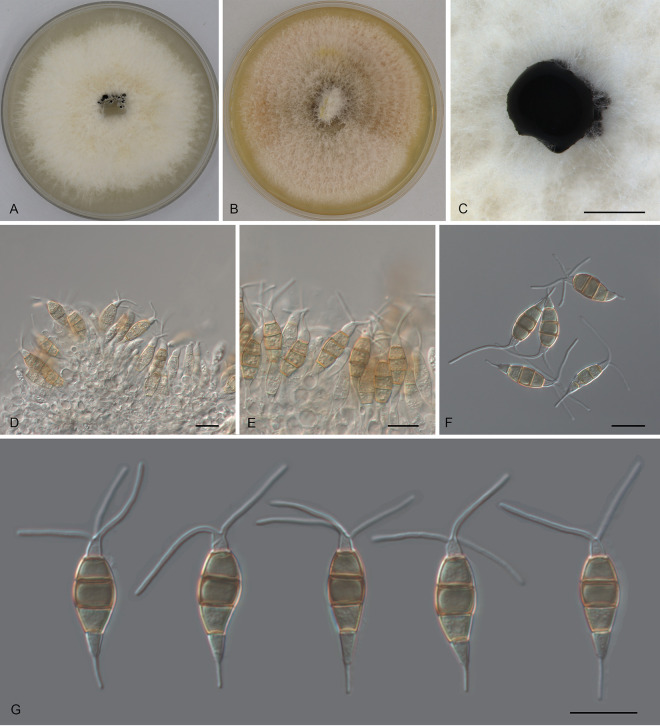
Morphology of *Pestalotiopsis lithocarpi* (CFCC 55100). (A) Colony on PDA after 10 days at 25°C; (B) colony on MEA after 10 days at 25°C; (C) conidioma formed on PDA; (D and E) conidiogenous cells giving rise to conidia; (F and G) conidia. Bars, 500 μm (C) and 10 μm (D to G).

*Pestalotiopsis lithocarpi* Ning Jiang, sp. nov. (Fig. 9). MycoBank number MB841312. Etymology: named after the host genus, *Lithocarpus*. Typus: China, Hainan Province, Changjiang Li Autonomous County, Bawangling National Forest Park, on diseased leaves of Lithocarpus chiungchungensis, 30 March 2019, Yong Li (holotype CAF 800025; ex-holotype culture CFCC 55100).

Conidiomata in culture sporodochial, solitary, erumpent, pulvinate, black, 450 to 1,100 μm in diameter, exuding black conidial masses. Conidiophores indistinct, usually reduced to conidiogenous cells. Conidiogenous cells hyaline, smooth, cylindrical to subcylindrical, annelidic, 4 to 10 by 3 to 5.5 μm, mean ± SD = 5.9 ± 2.8 by 4.2 ± 0.8 μm. Conidia fusoid, straight or slightly curved, 4-septate, smooth, slightly constricted at the septa, (17)18.5 to 21.5(23) by (5.5)6 to 7(8) μm, mean ± SD = 20.2 ± 1.4 by 6.6 ± 0.6 μm, L/W = 2.5 to 3.7; basal cell obconic with a truncate base, thin-walled, hyaline or pale brown, (3.5)4 to 5 μm; median cells 3, trapezoid or subcylindrical, concolorous, brown, thick-walled, the first median cell from base (3.5)4 to 5 μm long, the second cell 4 to 4.5(5) μm long, the third cell 4 to 5 μm long, together 12.5 to 14(14.5) μm long; apical cell conic with an acute apex, thin-walled, hyaline, (2.5)3 to 3.5 μm long; basal appendage unbranched, tubular, centric, straight or slightly bent, 2.5 to 4.5(5) μm long, mean ± SD = 3.6 ± 0.8 μm; apical appendages, 2 to 4 (mostly 3), unbranched, tubular, centric, straight or slightly bent, (9)12.5 to 21(24) μm long, mean ± SD = 16.7 ± 4.1 μm. Sexual morph unknown.

Colonies on MEA flat, spreading, with flocculent aerial mycelium forming concentric rings and entire edge, umber, reaching a 70-mm diameter after 10 days at 25°C, forming black conidiomata with black conidial masses; on PDA, flat, spreading, with flocculent aerial mycelium and feathery edge, white to pale luteous, reaching a 65-mm diameter after 10 days at 25°C, forming black conidiomata with black conidial masses.

Additional material examined, China, Hainan Province, Changjiang Li Autonomous County, Bawangling National Forest Park, on diseased leaves of *Lithocarpus chiungchungensis*, 30 March 2019, Yong Li (CFCC 55893).

Notes: Two isolates from leaf spots of *Lithocarpus chiungchungensis* clustered into a well-supported clade newly described here as *Pestalotiopsis lithocarpi* (Fig. 1). Phylogenetically, *P. lithocarpi* is close to P. dracontomelonis from diseased leaves of Dracontomelon dao (*Anacardiaceae*) collected in Thailand. *Pestalotiopsis lithocarpi* is similar to *P. dracontomelonis* in conidial size (17 to 23 by 5.5 to 8 μm in *Pestalotiopsis lithocarpi* versus 18 to 23 by 5.5 to 7.5 μm in *P. dracontomelonis*) (27). However, they can be distinguished by the length of the three median cells (12.5 to 14.5 μm in *P. lithocarpi* versus 13 to 17 μm in *P. dracontomelonis*) (27). In addition, *P. lithocarpi* can be distinguished from *P. dracontomelonis* by sequence data (nucleotide differences: in the ITS, 2/505 [0.4%], 1 insertion; in *tef1*, 5/457 [1.2%], 7 insertions).

*Pestalotiopsis lushanensis* F. Liu & L. Cai, Scientific Reports 7(no. 866):9 (2017).

Conidiomata in culture sporodochial, solitary, erumpent, pulvinate, black, 150 to 750 μm in diameter, exuding black conidial masses. Conidiophores indistinct, usually reduced to conidiogenous cells. Conidiogenous cells hyaline, smooth, cylindrical to subcylindrical, annelidic, 5.5 to 26.5 by 2.5 to 4.5 μm, mean ± SD = 13.6 ± 3.1 by 3.6 ± 0.9 μm. Conidia fusoid, straight or slightly curved, 4-septate, smooth, slightly constricted at the septa, (19.5)21 to 25(26.5) by (7)7.5 to 9(9.5) μm, mean ± SD = 23 ± 1.9 by 8.4 ± 0.6 μm, L/W = 2.3 to 3.5; basal cell obconic with a truncate base, thin-walled, hyaline or pale brown, (3.5)4 to 5.5(6.5) μm; median cells 3, trapezoid or subcylindrical, concolorous, brown, thick-walled, the first median cell from base (4)4.5 to 5.5(6) μm long, the second cell (4)4.5 to 5.5(6) μm long, the third cell (4.5)5 to 6(6.5) μm long, together (13)14 to 16.5(17.5) μm long; apical cell conic with an acute apex, thin-walled, hyaline, (2.5)3.5 to 4.5(5.5) μm long; basal appendage unbranched, tubular, centric, straight or slightly bent, (5.5)6 to 9(11) μm long, mean ± SD = 7.5 ± 1.4 μm; apical appendages, 3, unbranched, tubular, centric, straight or slightly bent, (10.5)15 to 22.5(26.5) μm long, mean ± SD = 18.6 ± 3.7 μm. Sexual morph unknown.

Colonies on MEA flat, spreading, with flocculent aerial mycelium forming concentric rings, radially folded surface and entire edge, white to isabelline, reaching a 70-mm diameter after 10 days at 25°C, forming black conidiomata with black conidial masses; on PDA, flat, spreading, with flocculent aerial mycelium forming concentric rings and entire edge, white to pale luteous, reaching a 70-mm diameter after 10 days at 25°C, forming black conidiomata with black conidial masses.

Materials examined: China, Guizhou Province, Zunyi City, Suiyang County, Kuankuoshui Natural Reserve, on diseased leaves of *Quercus serrata*, 23 November 2019, Dan-ran Bian (CFCC 54894).

Notes: A new isolate of *Pestalotiopsis lushanensis* was collected from Quercus serrata, which agrees well with the ex-type strain LC4344 in conidial dimension and characters (20 to 27 by 7.5 to 10 μm in LC4344) (18). Hence, *Quercus serrata* becomes a new host for *Pestalotiopsis lushanensis*, which was originally described from *Camellia* sp. (18).

*Pestalotiopsis nanjingensis* Qin Yang & He Li, Journal of Fungi 7(12, no. 1080):21 (2021).

Conidiomata in culture sporodochial, solitary, erumpent, pulvinate, black, 50 to 500 μm in diameter, exuding black conidial masses. Conidiophores indistinct, usually reduced to conidiogenous cells. Conidiogenous cells hyaline, smooth, cylindrical to subcylindrical, annelidic, 8.5 to 19 by 2 to 4.5 μm, mean ± SD = 13.4 ± 1.7 by 2.9 ± 0.9 μm. Conidia fusoid, straight or slightly curved, 4-septate, smooth, slightly constricted at the septa, (17.5)20 to 22(23.5) by (6.5)7 to 8.5(9) μm, mean ± SD = 20.8 ± 1 by 7.6 ± 0.7 μm, L/W = 2.2 to 3.5; basal cell obconic with a truncate base, thin-walled, hyaline or pale brown, (2.5)3 to 4.5(5) μm; median cells 3, trapezoid or subcylindrical, concolorous, brown, thick-walled, the first median cell from base 4 to 5(5.5) μm long, the second cell (4.5)5 to 5.5(6) μm long, the third cell (4)4.5 to 5.5(7) μm long, together (13)13.5 to 15.5(17.5) μm long; apical cell conic with an acute apex, thin-walled, hyaline, (2)3 to 4(4.5) μm long; basal appendage unbranched, tubular, centric, straight or slightly bent, (2)3.5 to 5(6.5) μm long, mean ± SD = 4.3 ± 0.9 μm; apical appendages, 2 or 3, unbranched, tubular, centric, straight or slightly bent, (8.5)11.5 to 16.5(20.5) μm long, mean ± SD = 14 ± 2.7 μm. Sexual morph unknown.

Colonies on MEA flat, spreading, with flocculent aerial mycelium and undulate edge, white to pale luteous, reaching a 60-mm diameter after 10 days at 25°C, forming black conidiomata with black conidial masses; on PDA, flat, spreading, with flocculent aerial mycelium forming concentric rings and undulate edge, white to pale gray, reaching a 70-mm diameter after 10 days at 25°C, forming black conidiomata with black conidial masses.

Material examined, China, Henan Province, Xinyang City, Shihe District, Jigong Mountain, on diseased leaves of *Quercus aliena*, 7 August 2019, Yong Li (CFCC 53882).

Notes: A new isolate of *Pestalotiopsis nanjingensis* was collected from *Quercus aliena*, which has ITS, *tef1*, and *tub2* sequences identical to those of the ex-type strain CSUFTCC16 (28). Hence, *Quercus aliena* becomes a new host for *Pestalotiopsis nanjingensis*, which was originally described from Camellia oleifera (28).

*Pestalotiopsis neolitseae* H. A. Ariyaw & K. D. Hyde, Mycosphere 9(5):1005 (2018).

Conidiomata in culture sporodochial, solitary, erumpent, pulvinate, black, 250 to 800 μm in diameter, exuding black conidial masses. Conidiophores indistinct, usually reduced to conidiogenous cells. Conidiogenous cells hyaline, smooth, cylindrical to subcylindrical, annelidic, 6 to 10 by 2.5 to 4 μm, mean ± SD = 7.7 ± 1.3 by 3.4 ± 0.6 μm. Conidia fusoid, straight or slightly curved, 4-septate, smooth, slightly constricted at the septa, (19)20 to 22.5(23.5) by 5.5 to 6.5(7) μm, mean ± SD = 21.2 ± 1.3 by 6.1 ± 0.4 μm, L/W = 2.8 to 4.1; basal cell obconic with a truncate base, thin-walled, hyaline or pale brown, 3.5 to 5(6) μm; median cells 3, trapezoid or subcylindrical, concolorous, brown, thick-walled, the first median cell from base 4.5 to 5 μm long, the second cell 4 to 5(5.5) μm long, the third cell 4.5 to 5 μm long, together 13 to 14.5 μm long; apical cell conic with an acute apex, thin-walled, hyaline, (2.5)3 to 4.5 μm long; basal appendage unbranched, tubular, centric, straight or slightly bent, (2)2.5 to 3.5 μm long, mean ± SD = 2.9 ± 0.6 μm; apical appendages, 2 or 3, unbranched, tubular, centric, straight or slightly bent, (9.5)11 to 14(14.5) μm long, mean ± SD = 12.6 ± 1.6 μm. Sexual morph unknown.

Colonies on MEA flat, spreading, with flocculent aerial mycelium forming concentric rings and undulate edge, white to pale gray, reaching a 30-mm diameter after 10 days at 25°C, sterile; on PDA, flat, spreading, with flocculent aerial mycelium and entire edge, white, reaching a 70-mm diameter after 10 days at 25°C, forming black conidiomata with black conidial masses.

Material examined, China, Hunan Province, Changsha City, Changsha Forest Botanical Garden, on diseased leaves of Lithocarpus amygdalifolius, 9 November 2020, Cheng-ming Tian and Ning Jiang (CFCC 54590).

Notes: A new isolate of *Pestalotiopsis neolitseae* was collected from *Lithocarpus amygdalifolius*, which agrees well with the type specimen in conidial dimension and characters (29). Hence, *Lithocarpus* becomes a new host genus for *Pestalotiopsis neolitseae*, which was originally described from Neolitsea villosa (*Lauraceae*).

*Pestalotiopsis rhodomyrtus* Yu Song, K. Geng, K. D. Hyde & Yong Wang bis, Phytotaxa 126(1): 27 (2013).

Conidiomata in culture sporodochial, solitary, erumpent, pulvinate, black, 100 to 450 μm in diameter, exuding black conidial masses. Conidiophores indistinct, usually reduced to conidiogenous cells. Conidiogenous cells hyaline, smooth, cylindrical to subcylindrical, annelidic, 2.5 to 6 by 2.5 to 4 μm, mean ± SD = 4.3 ± 1.1 by 2.9 ± 0.8 μm. Conidia fusoid, straight or slightly curved, 4-septate, smooth, slightly constricted at the septa, (20)21.5 to 24.5(27) by (6)6.5 to 7.5(8) μm, mean ± SD = 23 ± 1.7 by 7 ± 0.5 μm, L/W = 2.6 to 3.8; basal cell obconic with a truncate base, thin-walled, hyaline or pale brown, (3)3.5 to 5(6) μm; median cells 3, trapezoid or subcylindrical, concolorous, brown, thick-walled, the first median cell from base (4)4.5 to 6(6.5) μm long, the second cell (4.5)5 to 5.5(6) μm long, the third cell (4)4.5 to 5.5(6) μm long, together (13.5)14.5 to 16.5(18) μm long; apical cell conic with an acute apex, thin-walled, hyaline, (2.5)3 to 4.5(5) μm long; basal appendage unbranched, tubular, centric, straight or slightly bent, (2)3 to 5.5(7) μm long, mean ± SD = 4.3 ± 1.3 μm; apical appendages, 2 or 3, unbranched, tubular, centric, straight or slightly bent, (8)9.5 to 14.5(17.5) μm long, mean ± SD = 12 ± 2.4 μm. Sexual morph unknown.

Colonies on MEA flat, spreading, with flocculent aerial mycelium forming concentric rings and undulate edge, white to amber, reaching a 65-mm diameter after 10 days at 25°C, forming black conidiomata with black conidial masses; on PDA, flat, spreading, with flocculent aerial mycelium forming concentric rings and undulate edge, pale gray, reaching a 70-mm diameter after 10 days at 25°C, forming black conidiomata with black conidial masses.

Materials examined: China, Guizhou Province, Zunyi City, Suiyang County, Kuankuoshui Natural Reserve, on diseased leaves of Cyclobalanopsis augustinii, 23 November 2019, Dan-ran Bian (CFCC 55052); Shaanxi Province, Hanzhong City, Foping County, Dongshan Mountain, on diseased leaves of *Quercus aliena*, 7 September 2019, Yong Li (CFCC 54733).

Notes: Two new isolates of *Pestalotiopsis rhodomyrtus* were collected from Cyclobalanopsis augustinii and *Quercus aliena*, which agree with the type specimen in conidial dimension and characters (30). Hence, *Cyclobalanopsis augustinii* and *Quercus aliena* become new hosts for *Pestalotiopsis rhodomyrtus*, which was originally described from Rhodomyrtus tomentosa (*Myrtaceae*) (30).

**FIG 10:**
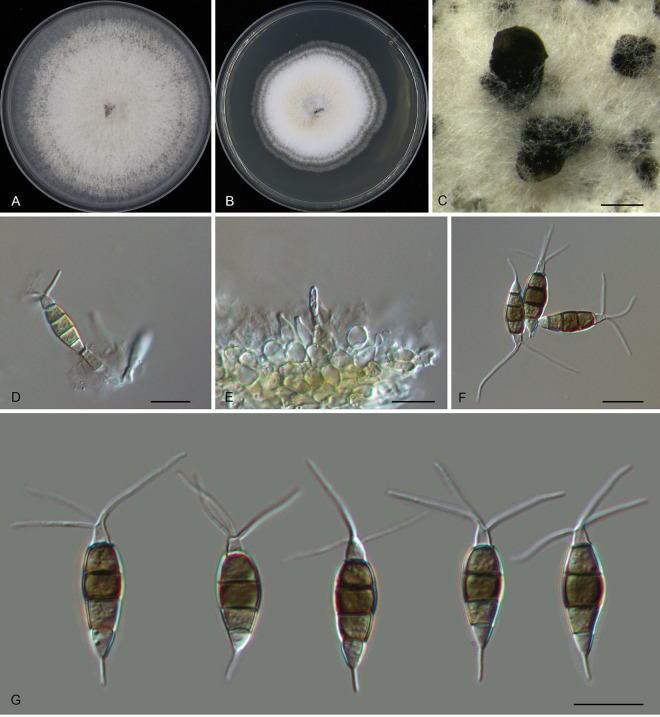
Morphology of *Pestalotiopsis shaanxiensis* (CFCC 54958). (A) Colony on PDA after 10 days at 25°C; (B) colony on MEA after 10 days at 25°C; (C) conidiomata formed on PDA; (D and E) conidiogenous cells giving rise to conidia; (F and G) conidia. Bars, 200 μm (C) and 10 μm (D to G).

Pestalotiopsis shaanxiensis Ning Jiang, sp. nov. (Fig. 10). MycoBank number MB843390. Etymology: named after the collection site of the type specimen, Shaanxi Province. Typus: China, Shaanxi Province, Hanzhong City, Foping County, Dongshan Mountain, on diseased leaves of *Quercus variabilis*, 7 September 2019, Yong Li (holotype CAF 800047; ex-holotype culture CFCC 54958).

Conidiomata in culture sporodochial, aggregated or solitary, erumpent, pulvinate, black, 100 to 550 μm in diameter, exuding black conidial masses. Conidiophores indistinct, usually reduced to conidiogenous cells. Conidiogenous cells hyaline, smooth, cylindrical to spherical, annelidic, 7 to 15 by 2 to 5.5 μm, mean ± SD = 10.5 ± 3 by 4.3 ± 1.1 μm. Conidia fusoid, straight or slightly curved, 4-septate, smooth, slightly constricted at the septa, (21)22 to 24.5(25) by (7)7.5 to 8.5(9) μm, mean ± SD = 23.1 ± 1.3 by 8 ± 0.4 μm, L/W = 2.5 to 3.3; basal cell obconic with a truncate base, thin-walled, hyaline or pale brown, (3.5)4 to 5(5.5) μm; median cells 3, trapezoid or subcylindrical, concolorous, pale brown to brown, thick-walled, the first median cell from base (4)4.5 to 5.5(6) μm long, the second cell (4.5)5 to 5.5(6) μm long, the third cell (4.5)5 to 5.5(6) μm long, together (13.5)14.5 to 16.5(17.5) μm long; apical cell conic with an acute apex, thin-walled, hyaline or pale brown, (3)3.5 to 4.5(5) μm long; basal appendage single, unbranched, tubular, centric, straight or slightly bent, (1.5)2.5 to 6(7.5) μm long, mean ± SD = 4.2 ± 1.6 μm; apical appendages, 3, unbranched, tubular, centric, straight or slightly bent, (13)13.5 to 18(22) μm long, mean ± SD = 15.6 ± 2.3 μm. Sexual morph unknown.

Colonies on MEA flat, spreading, with flocculent aerial mycelium forming concentric rings and undulate edge, white to pale luteous, reaching a 40-mm diameter after 10 days at 25°C, forming black conidiomata with black conidial masses; on PDA, flat, spreading, with flocculent aerial mycelium forming concentric rings and entire edge, white, reaching a 70-mm diameter after 10 days at 25°C, forming black conidiomata with black conidial masses.

Additional material examined, China, Shaanxi Province, Hanzhong City, Foping County, Dongshan Mountain, on diseased leaves of *Quercus variabilis*, 7 September 2019, Yong Li (CFCC 57356).

Notes: Two isolates of *Pestalotiopsis shaanxiensis* from *Quercus variabilis* formed a distinct clade phylogenetically close to P. biciliata and P. camelliae-oleiferae (Fig. 1). Morphologically, *P. shaanxiensis* has obviously wider conidia than *P. camelliae-oleiferae* (7 to 9 μm in *P. shaanxiensis* versus 5 to 7 μm in *P. camelliae-oleiferae*) (28). *P. shaanxiensis* can be distinguished from *P. biciliata* by one versus two basal appendages in the latter (16).

**FIG 11:**
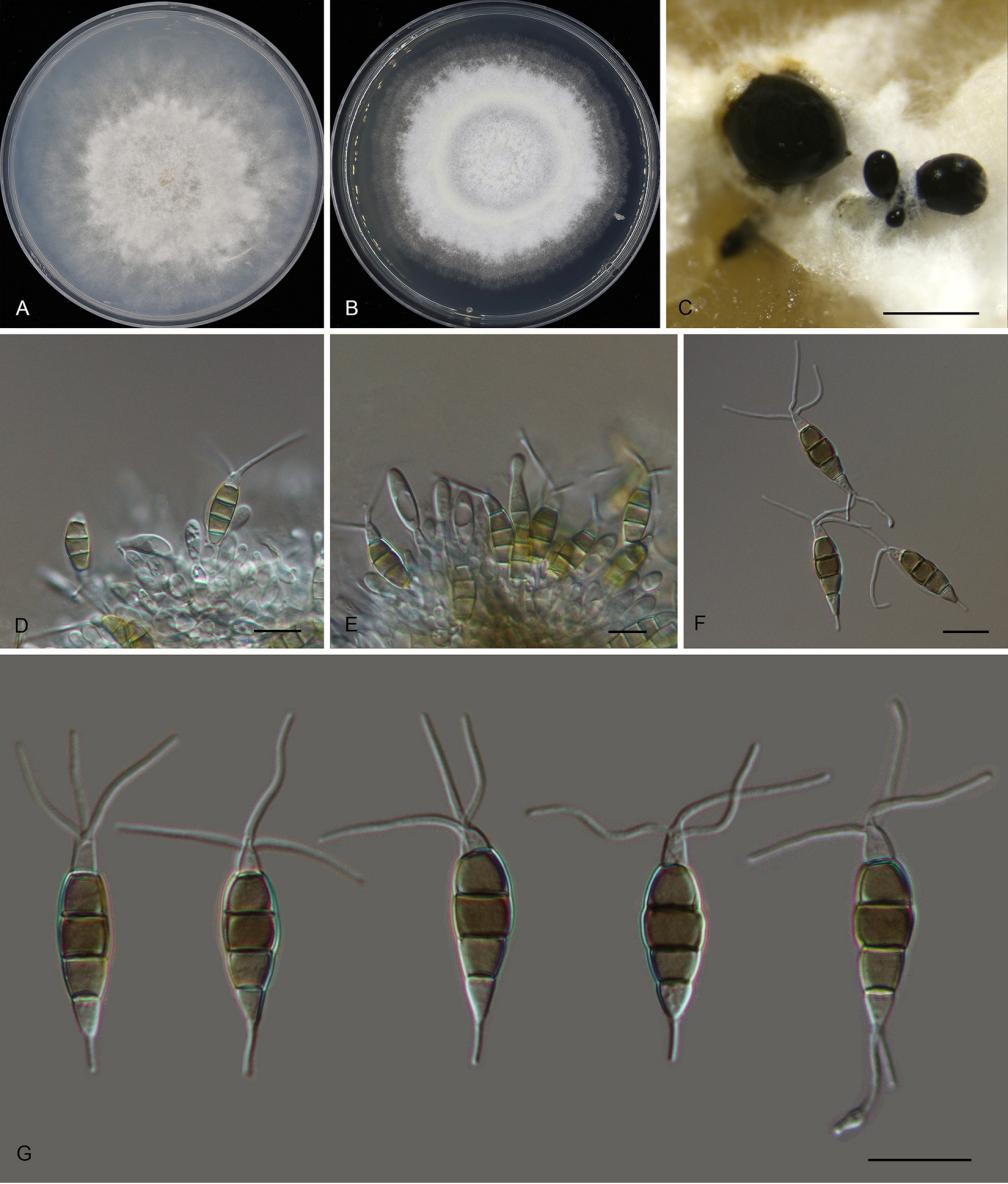
Morphology of *Pestalotiopsis silvicola* (CFCC 55296). (A) Colony on PDA after 10 days at 25°C; (B) colony on MEA after 10 days at 25°C; (C) conidiomata formed on PDA; (D and E) conidiogenous cells giving rise to conidia; (F and G) conidia. Bars, 300 μm (C) and 10 μm (D to G).

*Pestalotiopsis silvicola* Ning Jiang, sp. nov. (Fig. 11). MycoBank number MB843391. Etymology: *silva* = “forest” and *-cola* = “inhabiting”; in reference to its woody host. Typus: China, Hainan Province, Changjiang Li Autonomous County, Bawangling National Forest Park, on diseased leaves of Cyclobalanopsis kerrii, 30 March 2019, Yong Li (holotype CAF 800048; ex-holotype culture CFCC 55296).

Conidiomata in culture sporodochial, aggregated or solitary, erumpent, pulvinate, black, 50 to 450 μm in diameter, exuding black conidial masses. Conidiophores indistinct, usually reduced to conidiogenous cells. Conidiogenous cells hyaline, smooth, cylindrical to spherical, annelidic, 7.5 to 15 by 3 to 6 μm, mean ± SD = 10 ± 2.2 by 4 ± 1 μm. Conidia fusoid, straight or slightly curved, 4-septate, smooth, slightly constricted at the septa, (20)21 to 24(26) by (6)6.5 to 7.5(8.5) μm, mean ± SD = 22.5 ± 1.8 by 7 ± 0.7 μm, L/W = 2.6 to 3.8; basal cell obconic with a truncate base, thin-walled, hyaline or pale brown, (2.5)4 to 5.5(6) μm; median cells 3, trapezoid or subcylindrical, concolorous, brown, thick-walled, the first median cell from base (4)4.5 to 5 μm long, the second cell (3.5)4 to 5(6) μm long, the third cell 4 to 4.5(5.5) μm long, together (12)13 to 15(16) μm long; apical cell conic with an acute apex, thin-walled, hyaline, (3)4 to 5(5.5) μm long; basal appendages, 1 to 2, unbranched, tubular, centric, straight or slightly bent, (3.5)4.5 to 7(8) μm long, mean ± SD = 5.6 ± 1.1 μm; apical appendages, 3, unbranched, tubular, centric, straight or bent, (12)12.5 to 18.5(22.5) μm long, mean ± SD = 15.6 ± 2.9 μm. Sexual morph unknown.

Colonies on MEA flat, spreading, with flocculent aerial mycelium forming concentric rings and entire edge, white, reaching a 60-mm diameter after 10 days at 25°C, forming black conidiomata with black conidial masses; on PDA, flat, spreading, with flocculent aerial mycelium and undulate edge, white, reaching a 60-mm diameter after 10 days at 25°C, forming black conidiomata with black conidial masses.

Additional materials examined, China, Hainan Province, Changjiang Li Autonomous County, Bawangling National Forest Park, on diseased leaves of *Cyclobalanopsis kerrii*, 30 March 2019, Yong Li (CFCC 54915 and CFCC 57363).

Notes: Three isolates of *Pestalotiopsis silvicola* from *Cyclobalanopsis kerrii* clustered into a distinct clade phylogenetically close to P. aggestorum, P. colombiensis, and P. jinchanghensis (Fig. 1). Morphologically, they share similar conidial characters. However, *P. silvicola* can be distinguished by sequence data (nucleotide differences from *P. aggestorum*: in the ITS, 1/505 [0.2%]; in *tef1*, 1/464 [0.22%]; in *tub2*, 1/441 [0.23%]; from *P. colombiensis*: in the ITS, 2/505 [0.4%]; in *tef1*, 8/464 [1.72%]; in *tub2*, 2/441 [0.45%]; from *P. jinchanghensis*: in the ITS, 1/500 [0.2%]; in *tef1*, 18/464 [3.88%], 4-bp insertions; in *tub2*, 1/441 [0.23%]).

## DISCUSSION

In the present study, 43 isolates from *Fagaceae* leaf spots in China belonging to 16 *Pestalotiopsis* species were characterized. However, we investigated only 20 *Fagaceae* hosts of more than 320 reported species in China, and several provinces were not deeply investigated. Based on morphology and phylogeny, 10 of the 16 species described here have been proven to be new, indicating that many hidden *Pestalotiopsis* species may remain to be discovered from *Fagaceae* in the future.

Species of *Pestalotiopsis* are usually isolated from plant leaves (7, 8, 16, 18, 25). Two plurivorous species, *Pestalotiopsis kenyana* and P. monochaeta, were previously recorded from Castanea henryi and *C. mollissima* in China and from Quercus robur in the Netherlands, respectively (6, 16). These two species are not host-specific as they have been reported from various unrelated hosts, such as Camellia sinensis (*Theaceae*), *Coffea* sp. (*Rubiaceae*), and Taxus baccata (*Taxaceae*) (6, 16, 18). In the present study, 15 additional species are newly recorded from leaves of *Fagaceae* (Table 1), of which 10 are so far known only from fagaceous hosts.

Among pestalotioid species, appendages vary in number, origin, position, numbers of branches and the branching patterns (8). These characters have been proven appropriate and useful in delineating certain genera (8). For example, *Seimatosporium* is different from *Sporocadus* in that it forms appendages (9). However, *Pestalotiopsis changjiangensis* discovered in the present study is characterized by having only a single short, indistinct apical appendage, which is unique in *Pestalotiopsis* (Fig. 4).

This study revealed 16 *Pestalotiopsis* species associated with *Fagaceae* leaf spot symptoms in China. Further studies are now required, however, to confirm their pathogenicity.

## MATERIALS AND METHODS

### Sample collection and isolation.

Fresh specimens of diseased fagaceous leaves were collected from Anhui, Guangdong, Guangxi, Guizhou, Hainan, Henan, Hunan, Jiangxi, Shaanxi, and Sichuan Provinces in China from 2016 to 2021. A total of four host genera, 18 species, and one variety were investigated in the present study, *viz.*, *Castanopsis faberi*, *C. fissa*, *C. hainanensis*, *C. hystrix*, *C. lamontii*, *C. tonkinensis*, *Cyclobalanopsis augustinii*, *Cy. austrocochinchinensis*, *Cy. fleuryi*, *Cy. glauca*, *Cy. kerrii*, *Cy. neglecta*, *Lithocarpus amygdalifolius*, *L. chiungchungensis*, *Quercus aliena*, *Q. aliena* var. *acutiserrata*, *Q. griffithii*, *Q*. *serrata*, and *Q. variabilis*. The leaf samples were packed in paper bags and transferred to the laboratory for fungal isolation.

The leaf samples with typical spot symptoms were first surface sterilized for 1 min in 75% ethanol, 3 min in 1.25% sodium hypochlorite, and 1 min in 75% ethanol, then rinsed for 2 min in distilled water, and blotted on dry sterile filter paper. Then, the diseased areas of the leaves were cut into 0.5- by 0.5-cm pieces using an aseptic razor blade, transferred onto the surface of potato dextrose agar (PDA; 200 g potatoes, 20 g dextrose, 20 g agar per L) and malt extract agar (MEA; 30 g malt extract, 5 g mycological peptone, 15 g agar per L) plates, and incubated at 25°C to obtain fungal hyphae. Hyphal tips were then removed to new PDA plates to obtain pure cultures. The cultures were deposited in the China Forestry Culture Collection Center (CFCC; http://cfcc.caf.ac.cn/) and the specimens in the herbarium of the Chinese Academy of Forestry (CAF; http://museum.caf.ac.cn/).

### DNA extraction, sequencing, and phylogenetic analyses.

Genomic DNA was extracted from colonies grown on cellophane-covered PDA using a CTAB (cetyltrimethylammonium bromide) method (31). The amount of DNA was estimated by electrophoresis in 1% agarose gels, and the quality was measured using a NanoDrop 2000 instrument (Thermo Scientific, Waltham, MA, USA) following the user manual.

The following primer pairs were used for amplification of the gene regions sequenced in the present study: ITS1/ITS4 for the 5.8S nuclear ribosomal DNA gene with the two flanking internally transcribed spacer (ITS1 and ITS2) regions (32), EF1-728F/EF2 for the translation elongation factor 1-α (*tef1*) gene (33, 34), and T1/Bt2b and Bt2a/Bt2b for the beta-tubulin (*tub2*) gene (35, 36). The PCR conditions were set as follows: an initial denaturation step of 5 min at 94°C, followed by 35 cycles of 30 s at 94°C, 50 s at 52°C (ITS) or 54°C (*tef1* and *tub2*), and 1 min at 72°C, and a final elongation step of 7 min at 72°C. PCR amplification products were checked via electrophoresis in 2% agarose gels. DNA sequencing was performed using an ABI Prism 3730XL DNA analyzer with a BigDye Terminator kit v.3.1 (Invitrogen, USA) at the Shanghai Invitrogen Biological Technology Company Limited (Beijing, China).

The quality of the amplified nucleotide sequences was checked and the sequences assembled using SeqMan v.7.1.0. Reference sequences were retrieved from the National Center for Biotechnology Information (NCBI). Sequences were aligned using MAFFT v. 6 (37) and corrected manually using MEGA 6 (38). The phylogenetic analyses of the combined matrices were performed using maximum-likelihood (ML) and Bayesian inference (BI) methods. ML was implemented on the CIPRES Science Gateway portal (https://www.phylo.org) using RAxML-HPC BlackBox 8.2.10 (39), employing a GTRGAMMA substitution model with 1,000 bootstrap replicates, while BI was performed using a Markov chain Monte Carlo (MCMC) algorithm in MrBayes v. 3.0 (40). Two MCMC chains were run, starting from random trees, for 1,000,000 generations, and trees were sampled every 100th generation, resulting in a total of 10,000 trees. The first 25% of trees were discarded as burn-in of each analysis. Branches with significant Bayesian posterior probabilities (BPP) were estimated in the remaining 7,500 trees. Phylogenetic trees were viewed with FigTree v.1.3.1 and graphically processed by Adobe Illustrator CS5.

For closely related species with similar morphology, ITS, *tef1*, and *tub2* sequences of the respective species were compared pairwise. For this, the sequences of species pairs were aligned and the parts containing leading/trailing gaps were removed. Sequence differences of this alignment are recorded in the following way: number of nucleotide substitutions (excluding insertions and gaps)/total number of nucleotide characters, percent of sequence substitutions (in brackets), and the number of base pair insertions and gaps.

### Morphology.

The morphological data of the isolates collected in the present study were obtained from sporulating pure cultures grown on PDA or MEA in the dark at 25°C. The conidiomata were observed and photographed using a dissecting microscope (M205 C; Leica, Wetzlar, Germany). Microscope slides of conidiogenous cells and conidia were prepared in tap water, and the slides were examined and photographed with an Axio Imager 2 microscope (Zeiss, Oberkochen, Germany) equipped with an AxioCam 506 color camera or a Nikon Eclipse 80i microscope (Nikon, Tokyo, Japan) equipped with a Nikon digital sight DS-Ri2 camera, using differential interference contrast (DIC) illumination. For measurements, 50 conidia were randomly selected. Measurements of the conidia are reported as maximum and minimum in parentheses and the range representing the mean ± standard deviation of the measurements given in parentheses. Culture characteristics were recorded from 9-cm PDA or MEA plates after 10 days incubation at 25°C in the dark. To enable comparison of species growing on fagaceous hosts, available measurement data and sequence data are summarized in Table 1.

### Data availability.

The nucleotide sequence data from the present study were deposited in GenBank, and the accession numbers are listed in Table 2.

**TABLE 2:** Isolates and GenBank accession numbers used for phylogenetic analyses in this study^*a*^

Species	Isolate	Host/substrate	Origin	GenBank accession no.		
				ITS	*tub2*	*tef1*
*Neopestalotiopsis magna*	MFLUCC 12-0652^*b*^	*Pteridium* sp.	France	KF582795	KF582793	KF582791
*Pestalotiopsis abietis*	CFCC 53011^*b*^	*Abies fargesii*	China	MK397013	MK622280	MK622277
	CFCC 53012	*Abies fargesii*	China	MK397014	MK622281	MK622278
*P. adusta*	ICMP 6088^*b*^	Refrigerator door	Fiji	JX399006	JX399037	JX399070
	MFLUCC 10-146	*Syzygium* sp.	Thailand	JX399007	JX399038	JX399071
*P. aggestorum*	LC6301^*b*^	*Camellia sinensis*	China	KX895015	KX895348	KX895234
	LC8186	*Camellia sinensis*	China	KY464140	KY464160	KY464150
*P. anacardiacearum*	IFRDCC 2397^*b*^	Mangifera indica	China	KC247154	KC247155	KC247156
***P. anhuiensis***	**CFCC 54791** ^*b*^	***Cyclobalanopsis glauca***	**China**	**ON007028**	**ON005056**	**ON005045**
*P. arceuthobii*	CBS 434.65^*b*^	*Arceuthobium campylopodum*	USA	KM199341	KM199427	KM199516
*P. arenga*	CBS 331.92^*b*^	*Arenga undulatifolia*	Singapore	KM199340	KM199426	KM199515
*P. australasiae*	CBS 114126^*b*^	*Knightia* sp.	New Zealand	KM199297	KM199409	KM199499
	CBS 114141	*Protea* sp.	New South Wales	KM199298	KM199410	KM199501
*P. australis*	CBS 111503	*Protea neriifolia* × *susannae*	South Africa	KM199331	KM199382	KM199557
	CBS 114193^*b*^	*Grevillea* sp.	New South Wales	KM199332	KM199383	KM199475
*P. biciliata*	CBS 124463^*b*^	*Platanus* × *hispanica*	Slovakia	KM199308	KM199399	KM199505
	CBS 236.38	*Paeonia* sp.	Italy	KM199309	KM199401	KM199506
*P. brachiata*	LC2988^*b*^	*Camellia* sp.	China	KX894933	KX895265	KX895150
	LC8188	*Camellia* sp.	China	KY464142	KY464162	KY464152
	LC8189	*Camellia* sp.	China	KY464143	KY464163	KY464153
*P. brassicae*	CBS 170.26^*b*^	*Brassica napus*	New Zealand	KM199379	NA	KM199558
*P. camelliae*	MFLUCC 12-0277^*b*^	*Camellia japonica*	China	JX399010	JX399041	JX399074
*P. camelliae-oleiferae*	CSUFTCC08^*b*^	*Camelliae oleiferae*	China	OK493593	OK562368	OK507963
	CSUFTCC09	*Camelliae oleiferae*	China	OK493594	OK562369	OK507964
***P. castanopsidis***	**CFCC 54430** ^*b*^	***Castanopsis lamontii***	**China**	**OK339732**	**OK358508**	**OK358493**
	**CFCC 54305**	***Castanopsis hystrix***	**China**	**OK339733**	**OK358509**	**OK358494**
	**CFCC 54384**	***Castanopsis hystrix***	**China**	**OK339734**	**OK358510**	**OK358495**
*P. chamaeropis*	CBS 186.71^*b*^	*Chamaerops humilis*	Italy	KM199326	KM199391	KM199473
	LC3619	*Camellia* sp.	China	KX894991	KX895322	KX895208
	CFCC 55124	*Quercus acutissima*	China	OM746221	OM839894	OM839993
	CFCC 54977	*Quercus acutissima*	China	OM746223	OM839896	OM839995
	CFCC 55019	*Quercus aliena*	China	OM746224	OM839897	OM839996
	CFCC 55122	*Quercus aliena*	China	OM746229	OM839902	OM840001
	**CFCC 55023**	***Castanopsis fissa***	**China**	**OM746233**	**OM839906**	**OM840005**
	**CFCC 54776**	***Quercus variabilis***	**China**	**OM746234**	**OM839907**	**OM840006**
	**CFCC 55338**	***Quercus variabilis***	**China**	**OM746235**	**OM839908**	**OM840007**
***P. changjiangensis***	**CFCC 54314** ^*b*^	***Castanopsis tonkinensis***	**China**	**OK339739**	**OK358515**	**OK358500**
	**CFCC 54433**	***Castanopsis hainanensis***	**China**	**OK339740**	**OK358516**	**OK358501**
	**CFCC 52803**	***Cyclobalanopsis austrocochinchinensis***	**China**	**OK339741**	**OK358517**	**OK358502**
*P. clavata*	MFLUCC 12-0268^*b*^	*Buxus* sp.	China	JX398990	JX399025	JX399056
*P. colombiensis*	CBS 118553^*b*^	*Eucalyptus urograndis*	Colombia	KM199307	KM199421	KM199488
***P. cyclobalanopsidis***	**CFCC 54328** ^*b*^	***Cyclobalanopsis glauca***	**China**	**OK339735**	**OK358511**	**OK358496**
	**CFCC 55891**	***Cyclobalanopsis glauca***	**China**	**OK339736**	**OK358512**	**OK358497**
*P. digitalis*	MFLU 14-0208^*b*^	*Digitalis purpurea*	New Zealand	KP781879	KP781883	NA
*P. dilucida*	LC3232	*Camellia sinensis*	China	KX894961	KX895293	KX895178
*P. dilucida*	LC8184	*Camellia sinensis*	China	KY464138	KY464158	KY464148
*P. diploclisiae*	CBS 115449	*Psychotria tutcheri*	China	KM199314	KM199416	KM199485
	CBS 115587^*b*^	*Diploclisia glaucescens*	China	KM199320	KM199419	KM199486
*P. disseminata*	CBS 143904	*Persea americana*	New Zealand	MH554152	MH554825	MH554587
	MEAN 1165	Pinus pinea	Portugal	MT374687	MT374712	MT374699
*P. diversiseta*	MFLUCC 12-0287^*b*^	*Rhododendron* sp.	China	JX399009	JX399040	JX399073
*P. doitungensis*	MFLUCC 14-0115^*b*^	*Dendrobium* sp.	Thailand	MK993574	MK975837	MK975832
*P. dracaenicola*	MFLUCC 18-0913^*b*^	*Dracaena* sp.	Thailand	MN962731	MN962733	MN962732
*P. dracontomelonis*	MFLU 14-0207^*b*^	*Dracontomelon dao*	Thailand	KP781877	NA	KP781880
*P. ericacearum*	IFRDCC 2439^*b*^	*Rhododendron delavayi*	China	KC537807	KC537821	KC537814
*P. etonensis*	BRIP 66615^*b*^	*Sporobolus jacquemontii*	Australia	MK966339	MK977634	MK977635
*P. formosana*	NTUCC 17-009^*b*^	*Poaceae* sp.	China	MH809381	MH809385	MH809389
*P. furcata*	MFLUCC 12-0054^*b*^	*Camellia sinensis*	Thailand	JQ683724	JQ683708	JQ683740
	LC6691	*Camellia sinensis*	China	KX895030	KX895363	KX895248
***P. foliicola***	**CFCC 54440** ^*b*^	***Castanopsis faberi***	**China**	**ON007029**	**ON005057**	**ON005046**
	**CFCC 57359**	***Castanopsis faberi***	**China**	**ON007030**	**ON005058**	**ON005047**
	**CFCC 57360**	***Castanopsis faberi***	**China**	**ON007031**	**ON005059**	**ON005048**
*P. gaultheriae*	IFRD 411-014^*b*^	*Gaultheria forrestii*	China	KC537805	KC537819	KC537812
*P. gibbosa*	NOF 3175^*b*^	*Gaultheria shallon*	Canada	LC311589	LC311590	LC311591
*P. grevilleae*	CBS 114127^*b*^	*Grevillea* sp.	Australia	KM199300	KM199407	KM199504
***P. guangxiensis***	**CFCC 54308** ^*b*^	***Quercus griffithii***	**China**	**OK339737**	**OK358513**	**OK358498**
	**CFCC 54300**	***Quercus griffithii***	**China**	**OK339738**	**OK358514**	**OK358499**
***P. guizhouensis***	**CFCC 54803** ^*b*^	***Cyclobalanopsis glauca***	**China**	**ON007035**	**ON005063**	**ON005052**
	**CFCC 57364**	***Cyclobalanopsis glauca***	**China**	**ON007036**	**ON005064**	**ON005053**
*P. hawaiiensis*	CBS 114491^*b*^	*Leucospermum* sp.	USA	KM199339	KM199428	KM199514
*P. hispanica*	CBS 115391^*b*^	*Protea* sp.	Spain	MH553981	MH554640	MH554399
*P. hollandica*	CBS 265.33^*b*^	*Sciadopitys verticillata*	Netherlands	KM199328	KM199388	KM199481
*P. humicola*	CBS 336.97^*b*^	Soil	Papua New Guinea	KM199317	KM199420	KM199484
*P. hunanensis*	CSUFTCC15^*b*^	*Camellia oleifera*	China	OK493599	OK562374	OK507969
	CSUFTCC18	*Camellia oleifera*	China	OK493600	OK562375	OK507970
*P. inflexa*	MFLUCC 12-0270^*b*^	Unidentified tree	China	JX399008	JX399039	JX399072
P. intermedia	MFLUCC 12-0259^*b*^	Unidentified tree	China	JX398993	JX399028	JX399059
*P. italiana*	MFLU 14-0214^*b*^	*Cupressus glabra*	Italy	KP781878	KP781882	KP781881
*P. jesteri*	CBS 109350^*b*^	*Fragraea bodenii*	Papua New Guinea	KM199380	KM199468	KM199554
*P. jiangxiensis*	LC4399^*b*^	*Camellia* sp.	China	KX895009	KX895341	KX895227
*P. jinchanghensis*	LC6636^*b*^	*Camellia sinensis*	China	KX895028	KX895361	KX895247
	LC8190	*Camellia sinensis*	China	KY464144	KY464164	KY464154
*P. kaki*	KNU-PT-1804^*b*^	*Diospyros kaki*	Korea	LC552953	LC552954	LC553555
*P. kandelicola*	NCYU 19-0355^*b*^	*Kandelia candel*	China	MT560723	MT563100	MT563102
*P. kenyana*	CBS 442.67^*b*^	*Coffea* sp.	Kenya	KM199302	KM199395	KM199502
	LC6633	*Camellia sinensis*	China	KX895027	KX895360	KX895246
	**CFCC 54962**	***Quercus aliena***	**China**	**OM746237**	**OM839910**	**OM840009**
	**CFCC 55330**	***Cyclobalanopsis fleuryi***	**China**	**OM746238**	**OM839911**	**OM840010**
	**CFCC 54621**	***Quercus aliena* var. *acutiserrata***	**China**	**OM746239**	**OM839912**	**OM840011**
	**CFCC 54732**	***Cyclobalanopsis neglecta***	**China**	**OM746243**	**OM839916**	**OM840015**
	**CFCC 54742**	***Castanopsis hystrix***	**China**	**OM746245**	**OM839918**	**OM840017**
	**CFCC 54618**	***Quercus aliena* var. *acutiserrata***	**China**	**OM746248**	**OM839921**	**OM840020**
	**CFCC 54805**	***Cyclobalanopsis glauca***	**China**	**OM746253**	**OM839926**	**OM840025**
	**CFCC 55088**	***Castanopsis fissa***	**China**	**OM746254**	**OM839927**	**OM840026**
*P. knightiae*	CBS 111963	*Knightia* sp.	New Zealand	KM199311	KM199406	KM199495
	CBS 114138^*b*^	*Knightia* sp.	New Zealand	KM199310	KM199408	KM199497
*P. krabiensis*	MFLUCC 16-0260^*b*^	*Pandanus* sp.	Thailand	MH388360	MH412722	MH388395
*P. leucadendri*	CBS 121417^*b*^	*Leucadendron* sp.	South Africa	MH553987	MH554654	MH554412
*P. licualicola*	HGUP 4057^*b*^	*Licuala grandis*	China	KC492509	KC481683	KC481684
*P. lijiangensis*	CFCC 50738^*b*^	*Castanopsis carlesii* var. *spinulosa*	China	KU860520	KU844184	KU844185
*P. linearis*	MFLUCC 12-0271^*b*^	*Trachelospermum* sp.	China	JX398992	JX399027	JX399058
***P. lithocarpi***	**CFCC 55100** ^*b*^	***Lithocarpus chiungchungensis***	**China**	**OK339742**	**OK358518**	**OK358503**
	**CFCC 55893**	***Lithocarpus chiungchungensis***	**China**	**OK339743**	**OK358519**	**OK358504**
*P. lushanensis*	LC4344^*b*^	*Camellia* sp.	China	KX895005	KX895337	KX895223
	LC8182	*Camellia* sp.	China	KY464136	KY464156	KY464146
	LC8183	*Camellia* sp.	China	KY464137	KY464157	KY464147
	**CFCC 54894**	***Quercus serrata***	**China**	**OM746282**	**OM839955**	**OM840054**
*P. macadamiae*	BRIP 63738b	*Macadamia integrifolia*	Australia	KX186588	KX186680	KX186621
	BRIP 63739b	*Macadamia integrifolia*	Australia	KX186587	KX186679	KX186620
	BRIP 637441a	*Macadamia integrifolia*	Australia	KX186586	KX186678	KX186619
*P. malayana*	CBS 102220^*b*^	*Macaranga triloba*	Malaysia	KM199306	KM199411	KM199482
*P. monochaeta*	CBS 144.97^*b*^	Quercus robur	Netherlands	KM199327	KM199386	KM199479
	CBS 440.83	*Taxus baccata*	Netherlands	KM199329	KM199387	KM199480
***P. nanjingensis***	**CFCC 53882**	***Quercus aliena***	**China**	**OM746295**	**OM839968**	**OM840067**
	CSUFTCC16^*b*^	*Camellia oleifera*	China	OK493602	OK562377	OK507972
*P. nanningensis*	CSUFTCC10^*b*^	*Camellia oleifera*	China	OK493596	OK562371	OK507966
*P. neolitseae*	NTUCC 17-011^*b*^	*Neolitsea villosa*	China	MH809383	MH809387	MH809391
	**CFCC 54590**	***Lithocarpus amygdalifolius***	**China**	**OK339744**	**OK358520**	**OK358505**
*P. novae-hollandiae*	CBS 130973^*b*^	*Banksia grandis*	Australia	KM199337	KM199425	KM199511
*P. oryzae*	CBS 111522	*Telopea* sp.	USA	KM199294	KM199394	KM199493
	CBS 171.26	NA	Italy	KM199304	KM199397	KM199494
	CBS 353.69^*b*^	Oryza sativa	Denmark	KM199299	KM199398	KM199496
*P. pallidotheae*	MAFF 240993^*b*^	*Pieris japonica*	Japan	AB482220	NA	NA
*P. pandanicola*	MFLUCC 16-0255^*b*^	*Pandanus* sp.	Thailand	MH388361	MH412723	MH388396
*P. papuana*	CBS 331.96^*b*^	Coastal soil	Papua New Guinea	KM199321	KM199413	KM199491
	CBS 887.96	*Cocos nucifera*	Papua New Guinea	KM199318	KM199415	KM199492
*P. parva*	CBS 265.37	*Delonix regia*	NA	KM199312	KM199404	KM199508
	CBS 278.35^*b*^	*Delonix regia*	NA	KM199313	KM199405	KM199509
*P. photiniicola*	GZCC 16-0028^*b*^	*Photinia serrulata*	China	KY092404	KY047663	KY047662
*P. pini*	MEAN 1092	Pinus pinea	Portugal	MT374680	MT374705	MT374693
*P. pinicola*	KUMCC 19-0183^*b*^	*Pinus armandii*	China	MN412636	MN417507	MN417509
*P. portugalica*	CBS 393.48^*b*^	NA	Portugal	KM199335	KM199422	KM199510
*P. rhizophorae*	MFLUCC 17-0416^*b*^	*Rhizophora mucronata*	Thailand	MK764283	MK764349	MK764327
*P. rhododendri*	IFRDCC 2399^*b*^	*Rhododendron sinogrande*	China	KC537804	KC537818	KC537811
*P. rhodomyrtus*	LC4458	*Camellia sinensis*	China	KX895010	KX895342	KX895228
	HGUP4230^*b*^	*Rhodomyrtus tomentosa*	China	KF412648	KF412642	KF412645
	**CFCC 54733**	***Quercus aliena***	**China**	**OM746310**	**OM839983**	**OM840082**
	**CFCC 55052**	***Cyclobalanopsis augustinii***	**China**	**OM746311**	**OM839984**	**OM840083**
*P. rosea*	MFLUCC 12-0258^*b*^	*Pinus* sp.	China	JX399005	JX399036	JX399069
*P. scoparia*	CBS 176.25^*b*^	*Chamaecyparis* sp.	China	KM199330	KM199393	KM199478
*P. sequoiae*	MFLUCC 13-0399^*b*^	*Sequoia sempervirens*	Italy	KX572339	NA	NA
***P. shaanxiensis***	**CFCC 54958** ^*b*^	***Quercus variabilis***	**China**	**ON007026**	**ON005054**	**ON005043**
	**CFCC 57356**	***Quercus variabilis***	**China**	**ON007027**	**ON005055**	**ON005044**
***P. silvicola***	**CFCC 55296** ^*b*^	***Cyclobalanopsis kerrii***	**China**	**ON007032**	**ON005060**	**ON005049**
	**CFCC 54915**	***Cyclobalanopsis kerrii***	**China**	**ON007033**	**ON005061**	**ON005050**
	**CFCC 57363**	***Cyclobalanopsis kerrii***	**China**	**ON007034**	**ON005062**	**ON005051**
*P. spathulata*	CBS 356.86^*b*^	*Gevuina avellana*	Chile	KM199338	KM199423	KM199513
*P. spathuliappendiculata*	CBS 144035^*b*^	Phoenix canariensis	Australia	MH554172	MH554845	MH554607
*P. telopeae*	CBS 114137	*Protea* sp.	Australia	KM199301	KM199469	KM199559
	CBS 114161^*b*^	*Telopea* sp.	Australia	KM199296	KM199403	KM199500
	CBS 113606	*Telopea* sp.	Australia	KM199295	KM199402	KM199498
*P. terricola*	CBS 141.69^*b*^	Soil	Pacific Islands	MH554004	MH554680	MH554438
*P. thailandica*	MFLUCC 17-1616^*b*^	*Rhizophora mucronata*	Thailand	MK764285	MK764351	MK764329
*P. trachycarpicola*	OP068^*b*^	*Trachycarpus fortunei*	China	JQ845947	JQ845945	JQ845946
	IFRDCC 2403	*Podocarpus macrophyllus*	China	KC537809	KC537823	KC537816
	LC4523	*Camellia sinensis*	China	KX895011	KX895344	KX895230
*P. unicolor*	MFLUCC 12-0276^*b*^	*Rhododendron* sp.	China	JX398999	JX399030	NA
	MFLUCC 12-0275	Unidentified tree	China	JX398998	JX399029	JX399063
*P. verruculosa*	MFLUCC 12-0274^*b*^	*Rhododendron* sp.	China	JX398996	NA	JX399061
*P. yanglingensis*	LC4553^*b*^	*Camellia sinensis*	China	KX895012	KX895345	KX895231
	LC3412	*Camellia sinensis*	China	KX894980	KX895312	KX895197
*P. yunnanensis*	HMAS 96359^*b*^	*Podocarpus macrophyllus*	China	AY373375	NA	NA

a Isolates and sequences generated during the present study are in bold. NA, not available.

b Ex-type strain.
